# Targeting adenylate-forming enzymes with designed sulfonyladenosine inhibitors

**DOI:** 10.1038/s41429-019-0171-2

**Published:** 2019-04-15

**Authors:** Michaelyn C. Lux, Lisa C. Standke, Derek S. Tan

**Affiliations:** 1 Tri-Institutional PhD Program in Chemical Biology, Memorial Sloan Kettering Cancer Center, 1275 York Avenue, New York, NY 10065 USA; 2 Pharmacology Graduate Program, Weill Cornell Graduate School of Medical Sciences, Memorial Sloan Kettering Cancer Center, 1275 York Avenue, New York, NY 10065 USA; 3 Chemical Biology Program, Sloan Kettering Institute, and Tri-Institutional Research Program, Memorial Sloan Kettering Cancer Center, 1275 York Avenue, New York, NY 10065 USA

**Keywords:** Small molecules, Medicinal chemistry

## Abstract

Adenylate-forming enzymes are a mechanistic superfamily that are involved in diverse biochemical pathways. They catalyze ATP-dependent activation of carboxylic acid substrates as reactive acyl adenylate (acyl-AMP) intermediates and subsequent coupling to various nucleophiles to generate ester, thioester, and amide products. Inspired by natural products, acyl sulfonyladenosines (acyl-AMS) that mimic the tightly bound acyl-AMP reaction intermediates have been developed as potent inhibitors of adenylate-forming enzymes. This simple yet powerful inhibitor design platform has provided a wide range of biological probes as well as several therapeutic lead compounds. Herein, we provide an overview of the nine structural classes of adenylate-forming enzymes and examples of acyl-AMS inhibitors that have been developed for each.

## Introduction

Adenylate-forming enzymes are a mechanistic superfamily of structurally diverse enzymes that are found across all three domains of life and play critical roles in a wide range of biological processes, including protein synthesis [1, 2] and posttranslational modifications [3–6], nucleoside/nucleotide [7–9], amino acid [10, 11] and fatty acid metabolism [12–14], and natural product biosynthesis [15–24]. Accordingly, members of this superfamily are attractive targets for the development of biological probes and novel therapeutics in areas including infectious diseases [2, 6, 7, 17, 24–28], cancer [5, 10, 11], and neurodegenerative, metabolic, and autoimmune disorders [2].

Adenylate-forming enzymes generally catalyze a two-step reaction, first established by Berg in 1955 [29, 30]. The mechanism involves initial condensation of a carboxylic acid substrate with adenosine-5′-triphosphate (ATP) to form a reactive, tightly bound acyl adenylate (acyl-AMP) intermediate, followed by attack of a nucleophile on this mixed anhydride intermediate to form an ester, thioester, or amide product. Strikingly, at least nine different classes of enzymes comprising eight different protein folds are known to catalyze adenylation reactions, using distinct active-site residues and substrate-binding conformations.

Leveraging this mechanistic and structural information, work from a number of research groups has demonstrated that potent, selective inhibitors of adenylate-forming enzymes can be developed using acyl sulfonyladenosines (acyl-AMS), which mimic the tightly bound acyl-AMP reaction intermediate. Importantly, rational design of selective inhibitors can be achieved based on the structure of the carboxylic acid substrate, the binding orientation and active-site interactions of the tightly bound acyl-AMP intermediate, and the nature of the incoming nucleophile. Herein, we provide an overview of the adenylate-forming enzyme superfamily and the use of this general acyl-AMS platform to develop selective inhibitors of these enzymes.

## The adenylate-forming enzyme mechanistic superfamily

### Overview

**Fig. 1:**
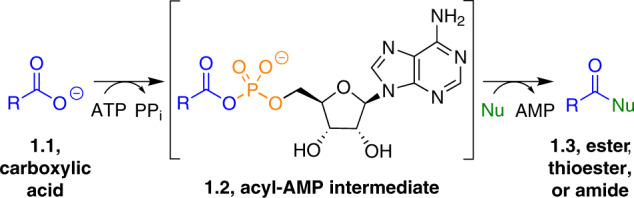
General mechanism for two half-reactions catalyzed by adenylate-forming enzymes. A carboxylic acid substrate (**1.1**) (blue) attacks ATP at the α-phosphate (orange) to form a reactive acyl-AMP (acyl adenylate) intermediate (**1.2**), which then reacts with a nucleophile (green) to form an ester, thioester, or amide product (**1.3**). In some cases, the second half-reaction is accompanied by a conformational change in the enzyme that leads to active-site remodeling. AMP adenosine-5′-*O*-monophosphate, ATP adenosine-5′-*O*-triphosphate

Adenylate-forming enzymes catalyze a wide range of coupling reactions between carboxylic acids and various nucleophiles, using both small molecule and protein substrates for each component [31]. In the first half-reaction, ATP is used to activate the carboxylic acid substrate (**1.1**), releasing pyrophosphate and forming a reactive acyl-AMP intermediate (**1.2**) (Fig. 1). Importantly, in the context of inhibitor design, this acyl-AMP intermediate generally remains tightly bound to the enzyme before catalysis of the second half-reaction. The acyl-AMP intermediate (**1.2**) then reacts with a nucleophile to form an ester, thioester, or amide product (**1.3**) with loss of AMP as the leaving group. In some cases, the second half-reaction is accompanied by a conformational change in the adenylate-forming enzyme to introduce new catalytic residues into the active site. Notably, there are a few biosynthetic pathways in which these two half-reactions are catalyzed by two separate enzymes [7, 32], although transfer of the acyl-AMP intermediate between the two enzymes must be rapid to avoid spontaneous hydrolysis or reactions with other nucleophiles [33, 34].

**Fig. 2:**
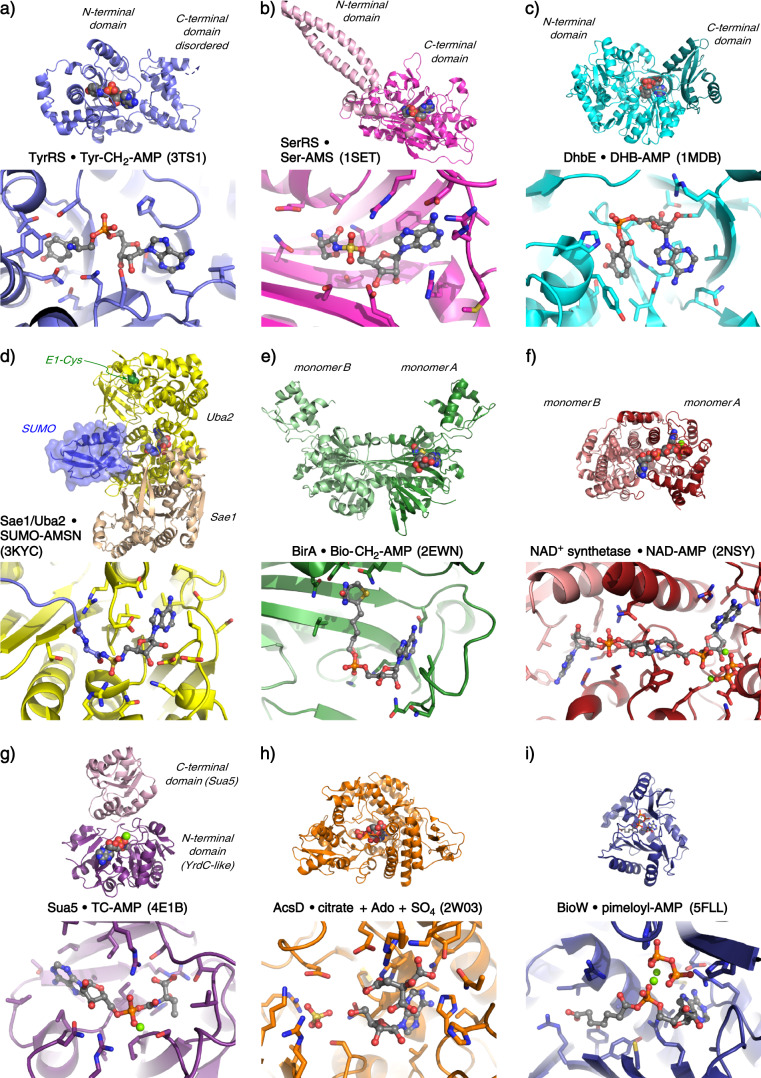
Structures of the nine classes of adenylate-forming enzymes. Top of **a**–**h**: Overall protein fold with acyl-AMP intermediate or mimic (spheres) bound. Bottom of **a**–**h**: Active site with acyl-AMP intermediate or mimic (ball-and-stick) bound; protein side chains within 4 Å of the ligand are shown (sticks); portions of structures that obscure the ligands are not shown. PDB ID codes are shown in parentheses. **a** Class I aminoacyl-tRNA synthetase *Bacillus stearothermophilus* tyrosyl-tRNA synthetase catalytic N-terminal domain with carbonyl-reduced intermediate mimic Tyr-CH_2_-AMP (tyrosinyl-AMP) (PDB ID: 3TS1) [44]. **b** Class II aminoacyl-tRNA synthetase *Thermus thermophilus* seryl-tRNA synthetase with adenylate mimic Ser-AMS (seryl-AMS) (PDB ID: 1SET) [134]. **c** ANL family enzyme *B. subtilis* DhbE with adenylate intermediate DHB-AMP (2,3-dihydroxybenzoyl-AMP) (PDB ID: 1MDB) [55]. **d** SUMO (small ubiquitin-like modifier) E1 activating enzyme human (Sae1/Uba2) with adenylate mimic SUMO-AMSN (SUMO1[T95C]-AMSN) (PDB ID: 3KYC) [60]. **e** Biotin protein ligase *Escherichia coli* BirA homodimer with carbonyl-reduced intermediate mimic Bio-CH_2_-AMP (biotinol-*O*-AMP) (PDB ID: 2EWN) [66]. **f** *N*-type ATP pyrophosphatase *B. subtilis* NAD^+^ synthetase homodimer with adenylate intermediate NAD-AMP (PDB ID: 2NSY) [68]. **g** YrdC-like carbamoyltransferase *Sulfolobus tokodaii* Sua5 with adenylate intermediate TC-AMP (threon-2-*N*-ylcarbamoyl-AMP) (PDB ID: 4E1B) [79, 80]. **h** NRPS-independent siderophore synthetase *Petrobacterium chrysanthemi* AcsD with substrate citrate and ATP fragments Ado (adenosine) and SO_4_ (sulfate) (PDB ID: 2W03) [91]. **i** BioW pimeloyl-CoA synthetase with adenylate intermediate pimeloyl-AMP (PDB ID: 5FLL) [40]. Abbreviations: AMP adenosine-5′-*O*-monophosphate, AMS adeonsine-5′-*O*-monosulfamate, AMSN 5′-(aminodeoxy)adenosine-5′-*N*-monosulfamide, ANL acyl-CoA synthetase/NRPS adenylation domain/luciferase, ATP adenosine-5′-*O*-triphosphate, CoA coenzyme A, NAD nicotinamide adenine dinucleotide, NRPS non-ribosomal peptide synthetase, tRNA transfer ribonucleic acid

In a striking example of parallel evolution, at least nine distinct protein classes comprising eight different structural folds are known to catalyze adenylation reactions: Class I aminoacyl-tRNA synthetases [1], Class II aminoacyl-tRNA synthetases [1], the ANL (acyl-CoA synthetase, non-ribosomal peptide synthetase (NRPS), luciferase) family [35], ubiquitin-family E1 activating enzymes [4, 36], biotin protein ligases (which share the same fold as Class II aminoacyl-tRNA synthetases but use a distinct active-site architecture) [37, 38], *N*-type ATP pyrophosphatases, YrdC-like carbamoyltransferases [9], NRPS-independent siderophore synthetases [24], and the recently described BioW acyl-CoA synthetases [39, 40] (Fig. 2 and Table 1). Remarkably, although each class of enzymes has distinct structural features, they all catalyze the same general biochemical transformation.

**Table 1:** Substrates of the nine classes of adenylate-forming enzymes

Enzyme class	Canonical acid	Canonical nucleophile(s)
Class I aminoacyl-tRNA synthetase	Amino acid	tRNA 3′-ribose (2′-OH)
Class II aminoacyl-tRNA synthetase	Amino acid	tRNA 3′-ribose (3′-OH)
ANL family	Acid	CoA-SH, CP-SH, luciferin-α-OOH
E1 activating enzyme	Ubl C terminus	E1-Cys-SH
Biotin protein ligase	Biotin	BCCP-Lys-NH_2_
*N*-type ATP pyrophosphatase	Acid	NH_3_
Yrd-like carbamoyltransferase	*N*-carboxy-Thr	tRNA-A^37^-6-NH_2_
NRPS-independent siderophore synthetase	Citric acid	R-NH_2_, R-OH
BioW acyl-CoA synthetase	Pimelate	CoA-SH

*ANL* acyl-CoA synthetase/NRPS adenylation domain/luciferase, *ATP* adenosine-5′-*O*-triphosphate, *BCCP* biotin carboxyl carrier protein subunit of acetyl-CoA carboxylase, *CoA* coenzyme A, *CP* peptidyl/acyl carrier protein, *E1* E1 activating enzyme, *NAD* nicotinamide adenine dinucleotide, *NRPS* non-ribosomal peptide synthetase, *Thr* threonine, *tRNA* transfer ribonucleic acid, *Ubl* ubiquitin-like modifier protein.

### Class I aminoacyl-tRNA synthetases

**Fig. 3:**
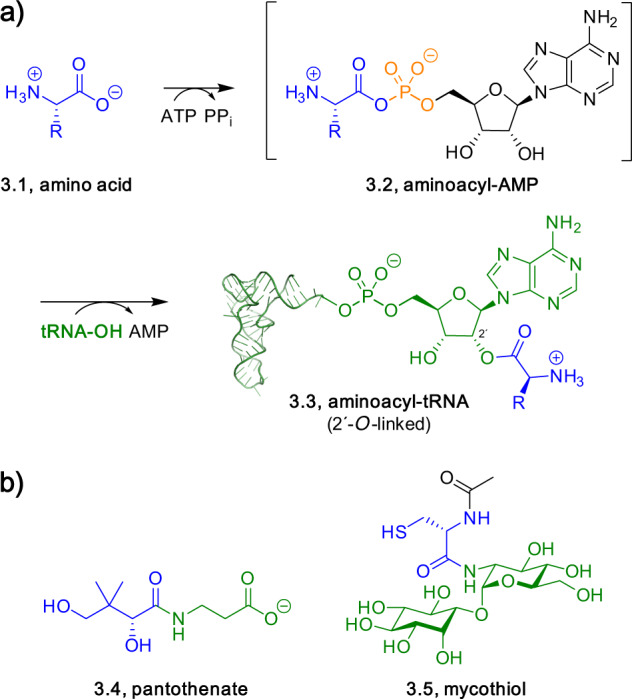
Biochemistry of class I aminoacyl-tRNA synthetases and related enzymes. **a** An amino acid (**3.1**) is adenylated to form an aminoacyl-AMP intermediate (**3.2**), which reacts with a tRNA 3′-ribose hydroxyl nucleophile (typically the 2′-hydroxyl) to form an aminoacyl-tRNA ester (**3.3**). **b** Pantothenate (**3.4**) and mycothiol (**3.5**) biosyntheses involve adenylate-forming enzymes having the same fold as class I aminoacyl-tRNA synthetases. Carboxylic acid substrate-derived fragments are shown in blue; nucleophile-derived fragments are shown in green. tRNA structure derived from PDB ID: 1GTR [213]

Aminoacyl-tRNA synthetases catalyze the activation of an amino acid (**3.1**) to form an aminoacyl-AMP intermediate (**3.2**), which then reacts with a ribose hydroxyl nucleophile at the 3′-end of the appropriate tRNA to form an aminoacyl-tRNA ester product (**3.3**) [26] (Fig. 3a). Two different classes of aminoacyl-tRNAs have been identified with distinct protein folds in the catalytic core [1, 41] (SCOPe c.26.1.1 and d.104.1.1) [42, 43], whereas various flanking domains are used to recognize the corresponding tRNA. Class I aminoacyl-tRNA synthetases have a nucleotide-binding Rossmann fold (Fig. 2a) and typically catalyze acylation of the 2′-hydroxyl of the tRNA 3′-ribose [1, 44, 45]. Of note in the context of inhibitor design, these enzymes bind the aminoacyl-AMP intermediate in an extended conformation. Interestingly, some of these enzymes have proofreading mechanisms that hydrolyze non-cognate aminoacyl-AMP intermediates and misacylated aminoacyl-tRNA products to prevent incorrect amino acid incorporation [46]. Further, bacterial pantothenate synthetase (PanC) [47, 48], which catalyzes coupling of pantoate to the amino group of β-alanine to form pantothenate (**3.4**), and mycobacterial cysteine ligase (MshC) [49], which catalyzes coupling of cysteine to the amino group of GlcN-Ins (1-d-*myo*-inosityl-2-amino-2-deoxy-α-d-glucopyranoside) in the biosynthesis of mycothiol (**3.5**), are structurally related to class I aminoacyl-tRNA synthetases (Fig. 3b).

### Class II aminoacyl-tRNA synthetases

**Fig. 4:**
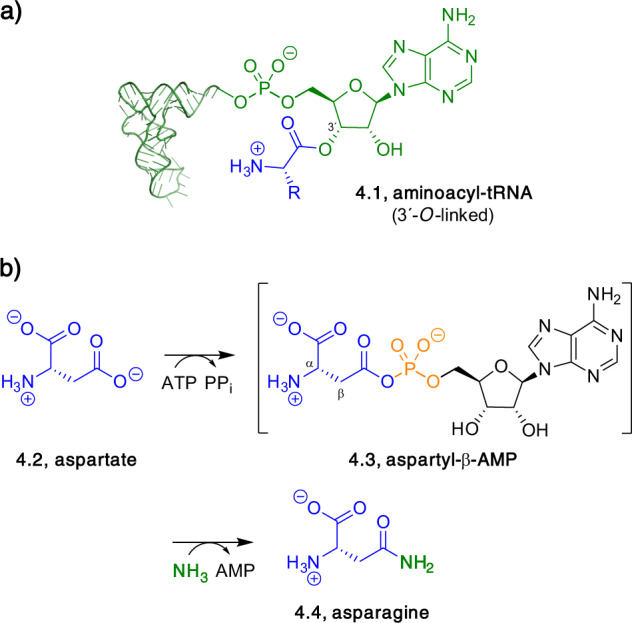
Biochemistry of class II aminoacyl-tRNA synthetases and related enzymes. **a** Aminoacyl-tRNA products (**4.1**) are produced by the same mechanism as in class I aminoacyl-tRNA synthetases (Fig. 3a), but are typically linked to the 3′-hydroxyl nucleophile of the tRNA 3′-ribose. **b** Asparagine synthetase A catalyzes adenylation of aspartate (**4.2**) at its β-carboxylate to form an aspartyl-β-AMP intermediate (**4.3**), which reacts with an ammonia nucleophile to form the primary carboxamide in asparagine (**4.4**). tRNA structure derived from PDB ID: 1ASY [214]

Class II aminoacyl-tRNA synthetases (SCOPe d.104.1.1) also catalyze activation of an amino acid to form an aminoacyl-AMP intermediate that is coupled with the corresponding tRNA 3′-ribose, but typically via the 3′-hydroxyl (**4.1**) [1] (Fig. 4a). The conserved catalytic core consists of a series of antiparallel β-sheets (Fig. 2b). In contrast to class I aminoacyl-tRNA synthetases, the aminoacyl-AMP intermediate is bound in a slightly bent conformation. Some class II enzymes also have proofreading mechanisms to prevent incorrect amino acid incorporation [46]. Prokaryotic asparagine synthetase A (AS-A) is structurally related to class II aminoacyl-tRNA synthetases and catalyzes activation of the aspartate side-chain carboxylate (**4.3**) and condensation with ammonia to form asparagine (**4.4**) [50] (Fig. 4b).

### ANL family enzymes

**Fig. 5:**
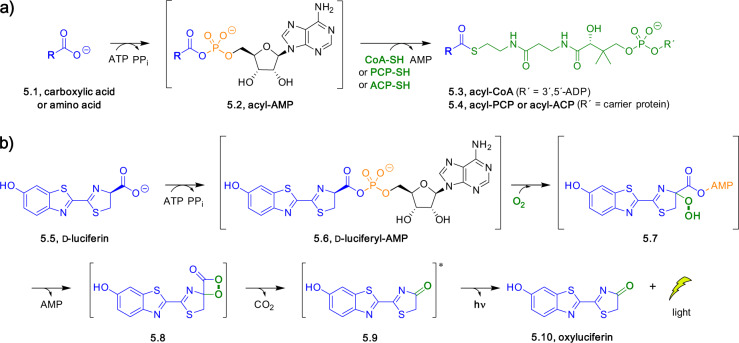
Biochemistry of ANL family enzymes. **a** Acyl-CoA synthetases and NRPS adenylation domains catalyze adenylation of carboxylic acids and amino acids (**5.1**) to form acyl-AMP intermediates (**5.2**), which react with a thiol nucleophile on the phosphopantetheine moiety of CoA or a carrier protein domain, respectively, to form thioester products (**5.3**, **5.4**). **b** Firefly luciferase catalyzes adenylation of d-luciferin (**5.5**) to form a luciferyl-AMP intermediate (**5.6**), which reacts via the corresponding enolate with molecular oxygen to form an α-peroxide intermediate (**5.7**). Cyclization of the intramolecular peroxide nucleophile forms a peroxylactone (**5.8**). Fragmentation forms excited oxyluciferin (**5.9**), which emits light and returns to the ground state (**5.10**). ACP acyl carrier protein, PCP peptidyl carrier protein

The ANL family (SCOPe e.23.1.1) [35] is comprised of acyl-CoA synthetases [51–53], NRPS adenylation domains [54, 55], and firefly luciferase [56, 57]. These enzymes catalyze adenylation of a variety of small-molecule carboxylic acids (**5.1**) to form acyl-AMP intermediates (**5.2**) (Fig. 5a). In the first two classes, this is followed by condensation with the thiol nucleophile of coenzyme A (**5.3**) or the analogous phosphopantetheine prosthetic group of a peptidyl or acyl carrier protein (ACP; thiolation) domain, respectively (**5.4**). The enzymes consist of a large N-terminal domain and a smaller C-terminal domain, which enclose the active site (Fig. 2c). Notably, the second half-reaction is characterized by a ≈140° rotation of the C-terminal domain, resulting in active-site remodeling [35]. Acyl-CoA synthetases are involved in metabolism of acetate as well as a wide range of fatty acid and other carboxylic acid substrates [12]. In contrast, NRPS adenylation domains activate amino acids and other carboxylic acid substrates during the biosynthesis of a wide range of bacterial natural products [15, 16]. In the third class, luciferase activates firefly d-luciferin (**5.5**) to form a d-luciferyl-AMP intermediate (**5.6**), which then reacts directly with molecular oxygen via the corresponding enolate to form an α-peroxide (**5.7**), which cyclizes to generate a four-membered α-peroxylactone intermediate (**5.8**) (Fig. 5b). Fragmentation of this intermediate leads to formation of oxyluciferin in an excited state (**5.9**), relaxation of which emits light [58]. Interestingly, luciferase can also activate the enantiomeric l-luciferin to form l-luciferyl-AMP, which condenses with CoA to form l-luciferyl-CoA, an inhibitor of the bioluminescence reaction. The luciferase acyl-CoA synthetase activity additionally has been suggested to have a role in conversion of natural l-cysteine-derived l-luciferin to the requisite d-luciferin substrate [58]. Recently, the OleC class of β-lactone synthetases, which catalyze cyclization of β-hydroxyacid substrates, was reported to fall in the ANL family based on sequence homology [23, 59]. Notably, a pair of enzymes, PtmA1 and PtmA2, which share the ANL family structure but appear to catalyze the adenylation and nucleophilic addition half-reactions separately, have been reported recently in the biosynthesis of platensimycin and platencin [32].

### Ubiquitin-family E1 activating enzymes

**Fig. 6:**
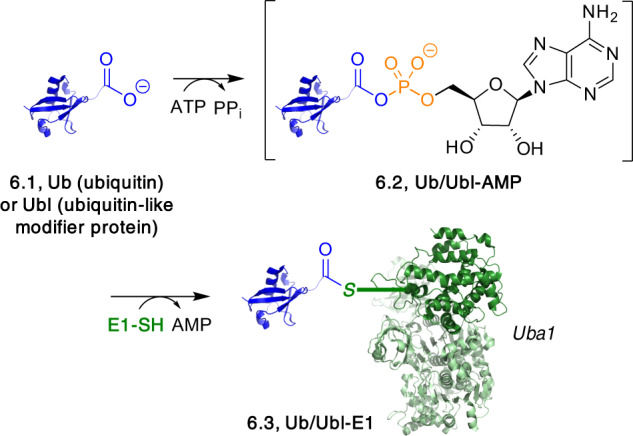
Biochemistry of ubiquitin-family E1 activating enzymes. The C-terminal carboxylate of Ub or a Ubl (**6.1**) is adenylated to form a Ub/Ubl-AMP intermediate (**6.2**), which reacts with a remote catalytic cysteine side-chain thiol nucleophile on the E1 enzyme to form a Ub/Ubl-E1 thioester conjugate (**6.3**). Ub and Ub E1 (Uba1) structures derived from PDB ID: 4NNJ [215]. Ub ubiquitin, Ubl ubiquitin-like modifier protein

E1 activating enzymes (SCOPe c.111.1.2) catalyze the first steps in the ubiquitin (Ub)/Ub-like modifier (Ubl) conjugation cascade [3–5, 36]. This involves initial adenylation of the C terminus of the Ub/Ubl (**6.1**), to form a Ub/Ubl-AMP intermediate (**6.2**), followed by condensation with the thiol nucleophile of a catalytic cysteine residue on the E1 enzyme itself to form a Ub/Ubl-E1 thioester product (**6.3**) (Fig. 6). This thioester intermediate then undergoes transthioesterification to the catalytic cysteine of an E2-conjugating enzyme. Terminal coupling of the Ub/Ubl to an amine nucleophile, typically a lysine side chain of a target protein, is then catalyzed by an E3 ligase, via another cysteine thioester intermediate in the case of HECT- and RBR-type E3s. Most notably, E1 activating enzymes use protein substrates for both the carboxylic acid and nucleophile components. E1 activating enzymes have a canyon-shaped active site with the base formed by two pseudosymmetric adenylation domains (one of which is inactive and in some cases a separate heterodimeric subunit) and the walls formed by a cysteine-containing domain and a ubiquitin-fold domain (Fig. 2d). The Ub/Ubl substrate binds at one end of the canyon and extends its C terminus across to the ATP-binding site at the other end. Notably, the second half-reaction requires a ≈130° rotation of the cysteine-containing domain coupled with remodeling of nearly half the residues in the active site [60]. Ub/Ubls are ubiquitous in eukaryotes, with polyubiquitination typically signaling for protein degradation, whereas monomeric acylation with Ub/Ubls serves as a more general posttranslational modification that impacts the function of the target protein.

**Fig. 7:**
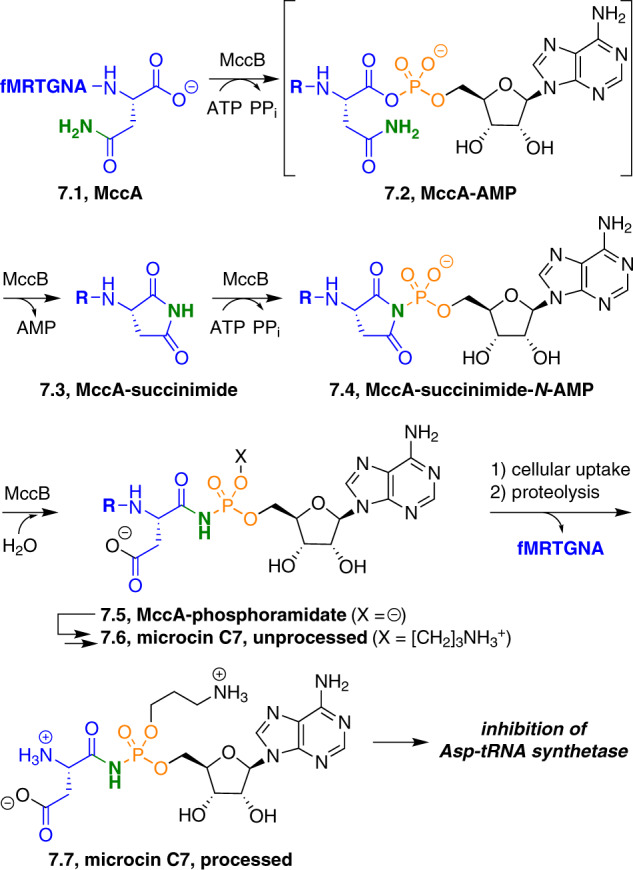
Biochemistry of *E. coli* microcin C7 synthetase MccB. The C-terminal carboxylate of the MccA precursor peptide (**7.1**) is adenylated to form a MccA-AMP intermediate (**7.2**), whose asparagine side-chain amide cyclizes to form a succinimide intermediate (**7.3**). MccB then catalyzes a second adenylation reaction to form a succinimide adenylate (**7.4**), which is hydrolyzed to form a phosphoramidate product (**7.5**). Downstream installation of an *O*-aminopropyl group provides microcin C (**7.6**). This Trojan horse antibiotic is taken up by target cells via peptide transporters, then the N-terminal peptide is proteolyzed to form an aspartyl-phosphoramidate (**7.7**), which inhibits aspartyl-tRNA synthetases in the target cell. R = fMRTGNA = *N*-formyl-Met-Arg-Thr-Gly-Asn-Ala peptide

Interestingly, although E1 activating enzymes are limited to eukaryotes, structurally related enzymes have been reported in bacteria. These enzymes lack the catalytic cysteine-containing domain used in the second half-reaction by E1 enzymes and instead use external nucleophiles. Examples include the *Escherichia coli* molybdenum cofactor biosynthetic enzyme MoeB, which catalyzes coupling of the C terminus of MoaD to a persulfide nucleophile [61], and the thiamin biosynthetic enzyme ThiF, which catalyzes coupling of the C terminus of ThiS to a similar persulfide nucleophile [62, 63]. Another related *E. coli* enzyme, MccB, activates the C-terminal asparagine of a peptide substrate MccA (**7.1**), leading to intramolecular attack of the amide side chain to form a succinimide intermediate (**7.3**) en route to the Trojan horse antibiotic microcin C7 (**7.6**) [64] (Fig. 7). After transporter-mediated uptake into target cells, the N-terminal peptide is proteolyzed to reveal an aspartyl-adenylate-mimetic phosphoramidate (**7.7**), which inhibits aspartyl-tRNA synthetase. Intriguingly, Severinov and colleagues [65] recently discovered a *Bacillus amyloliquefaciens* homolog of MccB that instead catalyzes cytidylation of the C terminus of an MccA-like peptide.

### Biotin protein ligases

**Fig. 8:**
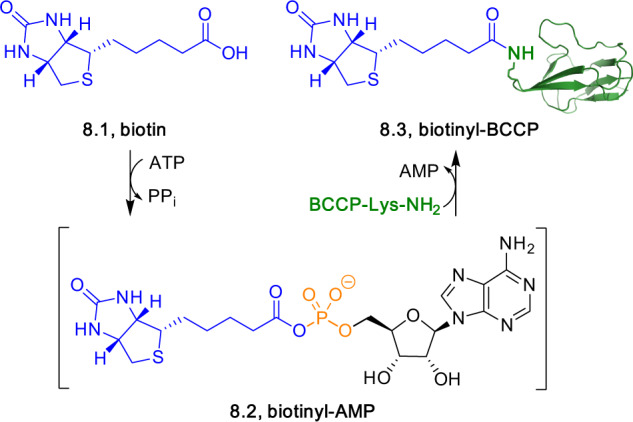
Biochemistry of biotin protein ligases. Biotin (**8.1**) is adenylated to form a biotinyl-AMP intermediate (**8.2**), which reacts with a lysine side-chain amine nucleophile on the BCCP subunit of acetyl-CoA carboxylase to form an amide product (**8.3**). In the case of the *E. coli* enzyme BirA, formation of the adenylate intermediate (**8.2**) also induces homodimerization of the enzyme, which binds to and represses the *bioO* biosynthetic operon for biotin. BCCP structure derived from PDB ID: 1BDO [216]. BCCP biotin carboxylate carrier protein

Biotin protein ligases (SCOPe b.34.1.1) activate biotin (**8.1**) to form a biotinyl-AMP intermediate (**8.2**), then couple it onto a lysine side chain of the biotin carboxylate carrier protein subunit of acetyl-CoA carboxylase (**8.3**) [6] (Fig. 8). A biotin carboxylase subunit then carboxylates this biotinyl group and the carboxyl group is subsequently transferred to acetyl-CoA by a carboxyltransferase subunit to form malonyl-CoA, the key precursor in fatty acid biosynthesis. The protein fold comprises five α-helix motifs and a mixed seven-stranded β-sheet [37, 66] (Fig. 2e). Interestingly, this fold is similar in overall structure to Class II aminoacyl-tRNA synthetases [38]. However, the binding mode of the acyl-AMP intermediate and the active-site residues are distinct (Fig. 2b), suggesting that these two classes may have diverged evolutionarily from a remote common progenitor or evolved convergently around this stable protein fold. In the case of the best characterized family member, BirA, the reaction intermediate biotinyl-AMP also functions as a co-repressor in transcription, allosterically activating dimerization of BirA, leading to binding to and repression of the biotin biosynthetic operon (*bioO*). Notably, fusion proteins of a BirA mutant that releases biotinyl-AMP prematurely have been used for proximity tagging of proteins in cells [33, 34].

### N-type ATP pyrophosphatases

**Fig. 9:**
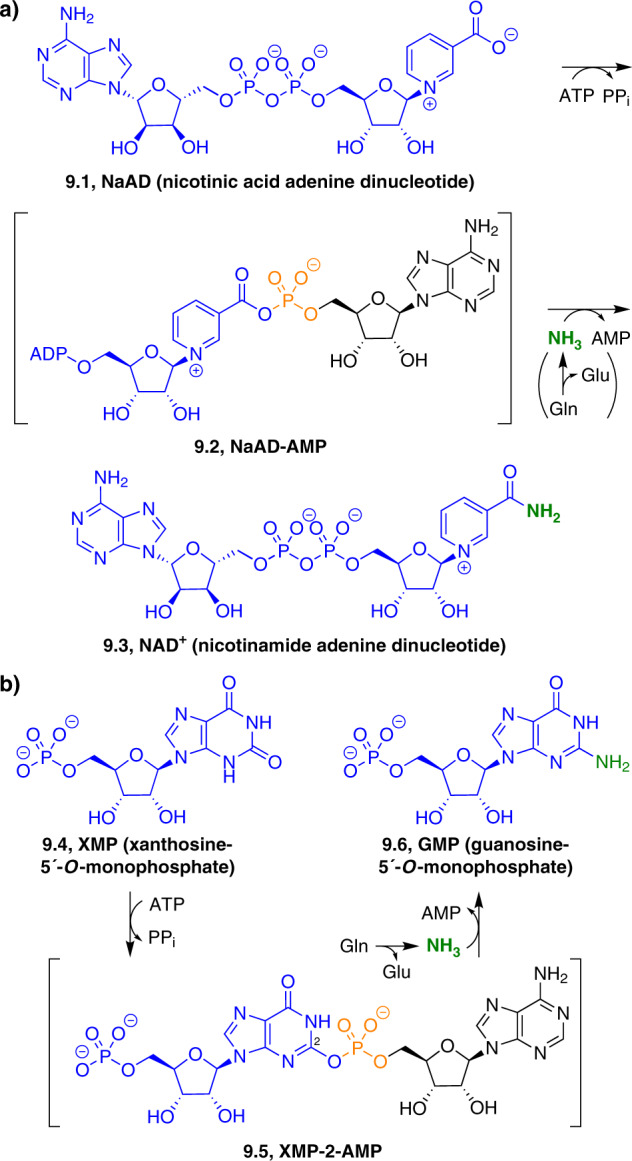
Biochemistry of N-type ATP pyrophosphatases. **a** In a canonical mechanism using a carboxylic acid substrate, NAD^+^ synthetase catalyzes adenylation of NaAD (**9.1**) to form a NaAD-AMP intermediate (**9.2**), which reacts with an ammonia nucleophile to form a NAD^+^ primary carboxamide product (**9.3**). The ammonia is typically, but not always, derived from hydrolysis of the side-chain amide of glutamine (Gln → Glu + NH_3_) by a separate glutamine amidotransferase domain or subunit. **b** In a mechanistic variant using a non-carboxylic acid substrate, GMP synthetase catalyzes adenylation of the urea oxygen of XMP (**9.4**) to form an XMP-2-AMP *O*-adenylyl isourea intermediate (**9.5**), which reacts with an ammonia nucleophile to form a GMP guanidine product (**9.6**). GMP guanosine-5′-*O*-monophosphate, NaAD nicotinic acid adenine dinucleotide, XMP xanthosine 5′-*O*-monophosphate

N-type ATP pyrophosphatases (SCOPe c.26.2.1) are a versatile family of enzymes involved in amino acid and nucleic acid metabolism [67–69]. The catalytic core consists of a five-strand parallel β-sheet flanked by α-helices, which catalyzes substrate adenylation and coupling to ammonia (Fig. 2f). The ammonia nucleophile is often supplied by hydrolysis of the side-chain amide of a glutamine co-substrate in a glutamine amidotransferase domain of the same enzyme or by a separate subunit. In canonical examples, the substrate is a carboxylic acid, as in the case of asparagine synthetase B (AS-B) [70] and NAD^+^ synthetase [68, 71] (Fig. 9a). However, a wide range of mechanistic variants are known to use other substrates, including the following: guanosine-5′-*O*-monophosphate (GMP) synthetase [67], which activates the C2-carbonyl oxygen of xanthosine 5′-monophosphate to form an *O*-adenylyl isourea (**9.5**), followed by substitution with ammonia to form GMP (**9.6**) (Fig. 9b); arginosuccinate synthetase [72, 73], which activates the side-chain amide of citrulline via its carbonyl oxygen and couples it to the α-amino group of aspartate during arginine biosynthesis; β-lactam synthetases [74–76], which catalyze an intramolecular amidation to form the characteristic β-lactam ring in these natural product antibiotics; and LarE [77], a sulfur insertase that couples a carboxylic acid substrate to a cysteine side chain during biosynthesis of a *Lactobacillus* cofactor.

### YrdC-like carbamoyltransferases

**Fig. 10:**
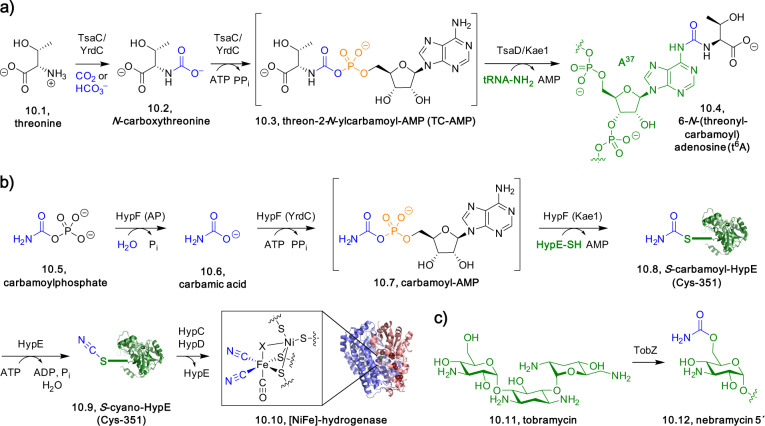
Biochemistry of YrdC-like carbamoyltransferases. **a** In t^6^A biosynthesis, TsaC/YrdC converts threonine (**10.1**) to *N*-carboxythreonine (**10.2**), then adenylates it to form a TC-AMP intermediate (**10.3**). A separate enzyme, TsaD/Kae1, condenses the adenylate with the 6-amino group of tRNA-A^37^ to form t^6^A (**10.4**). **b** In [NiFe]-hydrogenase maturation, the multidomain protein HypF carries out AP-domain-catalyzed hydrolysis of carbamoylphosphate (**10.5**) to carbamic acid (**10.6**), YrdC-like domain-catalyzed adenylation to form a carbamoyl-AMP intermediate (**10.7**) and Kae1-like domain-catalyzed condensation with the C-terminal Cys-351 side-chain thiol nucleophile of HypE to form an *S*-thiocarbamate intermediate (**10.8**). Downstream enzymes then catalyze dehydration to a thiocyanate intermediate (**10.9**), cyanide transfer to an iron center, and installation of the complex in the [NiFe]-hydrogenase active site (**10.10**, *X* = bridging ligand). **c** The natural product tobramycin (**10.11**) is *O*-carbamoylated by TobZ, which contains YrdC-like and Kae1-like domains, to form nebramycin 5′ (10.12). HypE structure derived from PDB ID: 3VTI [84]; [NiFe]-hydrogenase structure derived from PDB ID: 2FRV [217]. AP acyl phosphatase domain, t^6^A 6-*N*-(threon-2-*N*-ylcarbamoyl)adenosine, TC threon-2-*N*-ylcarbamoyl

YrdC-like carbamoyltransferases (SCOPe d.115.1.1) catalyze carbamoylation of a variety of substrates including nucleic acids, proteins, and small-molecule natural products [9]. The protein fold consists of a 7- to 11-strand mixed β-sheet with a distinctive ≈180° counterclockwise twist, surrounded by intervenening α-helical regions **(**Fig. 2g) [78–81]. These enzymes catalyze formation of carbamoyl-AMP intermediates, in contrast to the distinct transcarbamoylase (transcarbamylase) family, which uses carbamoylphosphate as an acyl donor [82]. Notably, condensation of the carbamoyl-AMP intermediate with a nucleophile in the second half-reaction is catalyzed by a separate Kae1-like domain or protein, with the adenylate intermediate thought to be shuttled between the two active sites. The most widely studied members of this family are involved in the biosynthesis of 6-*N*-(threon-2-*N*-ylcarbamoyl)adenosine (t^6^A), a tRNA modification found adjacent to anticodon sequences that recognize 5′-ANN-3′ codons and nearly universally conserved across all three domains of life [9]. Carbamoyltransferases in the Yrd/Sua5 family (renamed TsaC/Tcs1 in bacteria and TsaC2/Tcs2 in eukarya and archaea) initially condense the amino group of threonine (**10.1**) with CO_2_ or HCO_3_^–^ to form *N*-carboxythreonine (**10.2**), which is then adenylated to form threon-2-*N*-ylcarbamoyl-AMP (**10.3**) [83] (Fig. 10a). A second enzyme in the Kae1/YgjD/Qri7 family (renamed Tcs3/TsaD/Tcs4, respectively), operating as part of a larger protein complex, then transfers the acyl group to the 6-amino group of tRNA adenosine-37 to form the t^6^A product (**10.4**).

Another member of this family is the microbial [NiFe]-hydrogenase maturation protein HypF, which contains both YrdC-like and Kae1-like domains, as well as N-terminal acylphosphatase and Zn-finger domains [81, 84, 85]. Interestingly, this enzyme uses carbamoylphosphate (**10.5**) as the initial substrate, with several active sites linked by a long tunnel, allowing hydrolysis by the acylphosphatase domain to form carbamic acid (**10.6**), adenylation by the YrdC-like domain to form carbamoyl-AMP (**10.7**), and acyl transfer by the Kae1-like domain to the C-terminal Cys-351 side chain of a carbamoyl dehydratase HypE to form a thiocarbamate (**10.8**) (Fig. 10b). Subsequent HypE-catalyzed, ATP-dependent dehydration affords a thiocyanate intermediate (**10.9**), with the cyanide group ultimately transferred to an Fe center in the [NiFe]-hydrogenase (**10.10**) by downstream HypC and HypD enzymes. Related enzymes in the CmcH/NodU family catalyze *O*-carbamoylation of small-molecule natural products, including cephamycin (CmcH), rhizobial nodulation factors (NodU), novobiocin (NovN), and tobramycin (TobZ) (Fig. 10c) [80]. Notably, the order of the YrdC-like and Kae1-like domains is reversed in TobZ compared with HypF.

### NRPS-independent siderophore synthetases

**Fig. 11:**
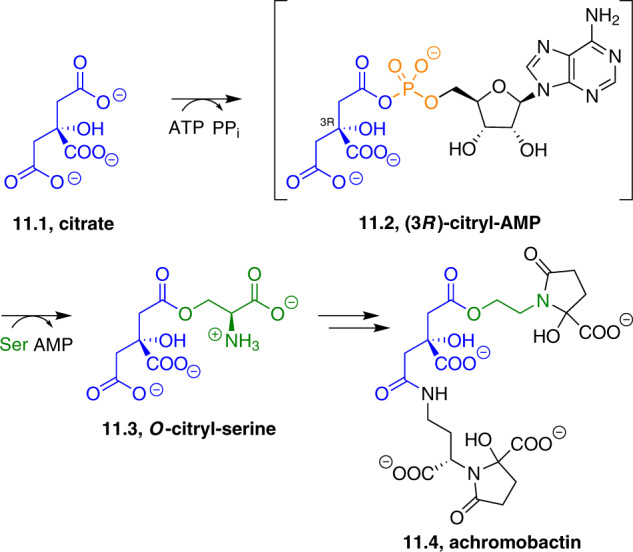
Biochemistry of NRPS-independent siderophore synthetases. This family typically uses diacid or monofunctionalized derivatives of diacids as substrates and couples them to alcohol or amine nucleophiles. *P. crysanthemi* AcsD catalyzes desymmetrizing adenylation of citrate (**11.1**) to form a (3*R*)-citryl-AMP intermediate (**11.2**), which reacts with a serine side-chain hydroxyl nucleophile to form an ester product (**11.3**), en route to the siderophore achromobactin (**11.4**)

Siderophores are iron-chelating natural products that are used by pathogenic bacteria to acquire iron from their hosts [28, 86, 87]. Most are produced by hybrid NRPS-polyketide biosynthetic pathways [88]. However, some siderophores have been found to be produced by distinct pathways involving NRPS-independent siderophore synthetases [24, 89, 90]. The enzyme family was initially discovered in studies of the biosynthesis of aerobactin and typically catalyzes adenylation of diacid substrates or their derivatives, followed by coupling to amine or alcohol nucleophiles. These enzymes can carry out desymmetrization and macrocyclization reactions. Structural studies of AcsD, which catalyzes enantioselective adenylation of citrate (**11.2**) in the biosynthesis of achromobactin (**11.4**), revealed a novel protein fold [91, 92] (Fig. 11). The overall structure comprised three domains resembling a thumb, palm, and fingers that surround the active site (Fig. 2g). Additional NRPS-independent siderophore synthetases have been identified in biosynthetic pathways of other siderophores, including aerobactin, alcaligin, anthrachelin, legiobactin, petrobactin, staphyloferrins A and B, rhizoferrins, and vibrioferrin [24, 89, 90]. Several of these enzymes have been characterized structurally, including AsbB (petrobactin) [93], IucA and IucC (aerobactin) [94, 95], and AlcC (alcaligin, putative) [96].

### BioW acyl-CoA synthetases

**Fig. 12:**
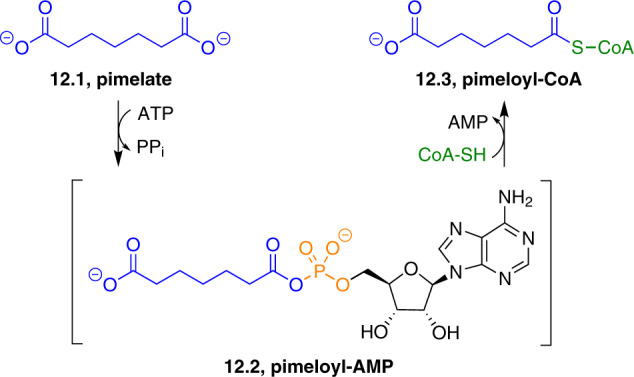
Biochemistry of BioW acyl-CoA synthetases. Pimelate (**12.1**) is adenylated to form a pimeloyl-AMP intermediate (**12.2**), which reacts with a CoA thiol nucleophile to form a pimeloyl-CoA thioester product (**12.3**), en route to biotin

Recently, a ninth class of adenylate-forming enzymes was discovered. The pimeloyl-CoA synthetase BioW comprises a new catalytic fold for adenylate-forming enzymes, with the active site sandwiched between a small N-terminal domain and a larger C-terminal domain [39, 40] (Fig. 2h). It catalyzes activation of pimelate (**12.1**) to form pimeloyl-AMP (**12.2**), followed by thioesterification with CoA to form pimeloyl-CoA (**12.3**), an intermediate in biotin biosynthesis (Fig. 12). Interestingly, this enzyme has been shown to be capable of proofreading, similar to aminoacyl-tRNA synthetases, by hydrolysis of non-cognate adenylate intermediates.

### Natural product-inspired design of adenylate-forming enzyme inhibitors

**Fig. 13:**
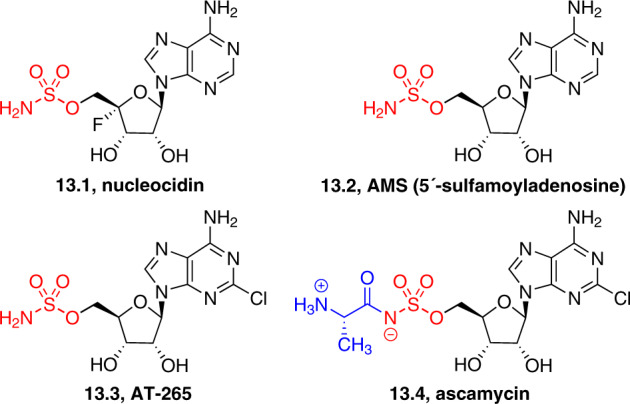
Sulfamoyladenosine (AMS) natural products

Natural products have provided essential inspiration for the development of inhibitors of adenylate-forming enzymes. In particular, the sulfamoyladenosines are a small family of natural products that contain a unique sulfamate moiety (Fig. 13). The first member of this class, nucleocidin (**13.1**), was reported in 1957 by researchers at American Cyanamid as a potent antitrypanosomal [97, 98], although its complete structure was not elucidated until 1969 [99]. Its mechanism of action was originally proposed to involve inhibition of protein synthesis [100], although more recent work has implicated inhibition of ubiquitin-family E1 activating enzymes as another potential mechanism [101]. The desfluorinated analogue AMS (**13.2**) has been studied extensively [102, 103] as a more readily synthesized analogue [104, 105] and has also been isolated as a natural product in its own right in 1986 [106]. Another close analogue, AT-265 (**13.3**), was isolated in 1982 [107].

A novel family member, ascamycin (**13.4**), was isolated in 1984 as an antibiotic that also inhibits protein synthesis but is more selective than AT-265 [108]. Interestingly, bacteria sensitive to ascamycin were found to dealanate the natural product to form AT-265, the presumed active species [109].

In pioneering work, Ishida and colleagues [110] recognized that ascamycin was also a close analogue of alanyl-AMP, the reaction intermediate formed by alanyl-tRNA synthetase. As no co-crystal structures of aminoacyl-tRNA synthetases with their cognate aminoacyl-AMP intermediates had yet been reported, they posited that the acyl sulfamate moiety could act as a stable, non-hydrolyzable bioisostere of the labile acyl phosphate. Thus, they synthesized alanyl-AMS and showed that it is a competitive inhibitor of alanyl-tRNA synthetase. As a result, numerous co-crystal structures of aminoacyl-tRNA synthetases with aminoacyl-AMS inhibitors were reported (vide infra). Based on this seminal discovery, the acyl-AMS inhibitor design platform has subsequently been expanded to a wide range of other adenylate-forming enzymes.

## Rational design of acyl-AMS inhibitors of adenylate-forming enzymes

**Fig. 14:**
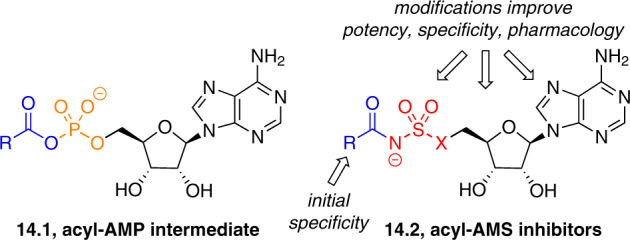
General acyl-AMS inhibitor design platform. Acyl-AMP intermediates (**14.1**) are generally bound tightly by the corresponding adenylate-forming enzymes and can be mimicked with non-hydrolyzable acyl-AMS analogues (14.2, *X* = O or NR). Initial specificity for the desired adenylate-forming enzyme is provided by the acyl group (blue) and additional modifications can be made in the acyl, sulfamate (red), ribose, and adenine regions in analogues

The seminal work of Ishida and colleagues [110] demonstrated that alanyl-AMS could be used to inhibit an alanyl-tRNA synthetase. As adenylate-forming enzymes typically bind their cognate acyl-AMP reaction intermediates (**14.1**) tightly between the adenylation and acylation half-reactions, this suggested that the acyl-AMS (**14.2**) inhibitor design strategy could be applied broadly to other members of the mechanistic superfamily (Fig. 14). Indeed, several research groups have used this approach effectively to target six out of the nine classes of adenylate-forming enzymes. Typically, the identity of the acyl group provides substantial selectivity for the targeted enzyme, while modifications to the sulfamate, ribose, and adenine motifs can be used to modulate potency, specificity, and pharmacological properties.

### Inhibitors of class I aminoacyl-tRNA synthetases and related enzymes

**Fig. 15:**
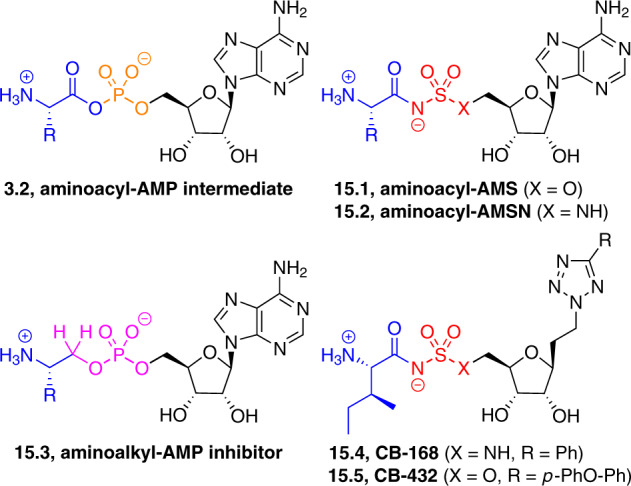
Inhibition of Class I aminoacyl-tRNA synthetases. Structures of aminoacyl-AMP reaction intermediates (**3.2**) and related adenylate-mimetic inhibitors (**15.1**–**15.5**)

Following the precedent set by Ishida and colleagues [110] above, a number of class I aminoacyl-tRNA synthetases have been co-crystallized with aminoacyl-AMS analogues (**15.1**), or the corresponding sulfamides (**15.2**), that mimic the cognate aminoacyl-AMP intermediate (**3.2**) [111–117] (Fig. 15). Notably, carbonyl-reduced aminoalkyl-AMP analogues (**15.3**) have also been used as non-hydrolyzable mimics of the adenylate intermediate, but tend to be more effective against class I aa-tRNA synthetases than class II aa-tRNA synthetases [118]. In most class I enzymes, the carbonyl group of the aminoacyl-AMP intermediate does not interact with active-site residues, consistent with its dispensability for binding. In contrast, in class II enzymes, this carbonyl interacts with a conserved arginine side chain, consistent with the decreased affinity of the carbonyl-reduced analogues in these cases.

In addition to the fundamental mechanistic interest in aminoacyl-tRNA synthetases, these enzymes are implicated in a wide range of human diseases [2] and have attracted particular interest as antibacterial targets [25]. However, achieving selective inhibition of a bacterial aminoacyl-tRNA synthetase over the corresponding human enzyme presents a significant challenge. Along these lines, researchers at Cubist Pharmaceuticals found that replacement of the adenine moiety in isoleucyl-AMS with heterocyclic motifs (**15.4**, CB-168; **15.5**, CB-432) provided over two-log selectivity for bacterial isoleucyl-tRNA synthetases over the human enzyme [119, 120] (Fig. 15). Further, CB-432 exhibited in vitro antibacterial activity and in vivo efficacy in a mouse model of *Streptomyces pyogenes* infection. However, high serum protein binding precluded further development of this compound. It has been noted that the zwitterionic character of aminoacyl-AMS inhibitors may limit their cell permeability and other pharmacological properties [25]. To address this problem, dipeptidyl-AMS prodrugs (not shown) have been investigated as antibacterials postulated to be taken up by peptide transporters then cleaved by intracellular peptidases to release the parent aminoacyl-AMS inhibitors [121, 122]. In addition, analogues of aminoacyl-AMS inhibitors have been investigated as treatments for psoriasis [123] and as immunosuppressants [124].

Intriguingly, Lee and colleagues [125–127] have recently developed analogues of leucyl-AMS (not shown) as potential anticancer agents. However, rather than inhibiting the catalytic activity of leucyl-tRNA synthetase, these analogues selectively block a second, non-catalytic function, in which the protein acts as a leucine-sensing GTPase activating protein for Rag GTPase, leading to activation of the mTORC1 complex that regulates protein translation and cell growth [128, 129]. Although the parent inhibitor leucyl-AMS exhibited nonspecific cytotoxicity against both cancer and normal cell lines, two analogues had selective activity against several cancer cell lines, presumably by selectively targeting this second, non-catalytic function [125].

**Fig. 16:**
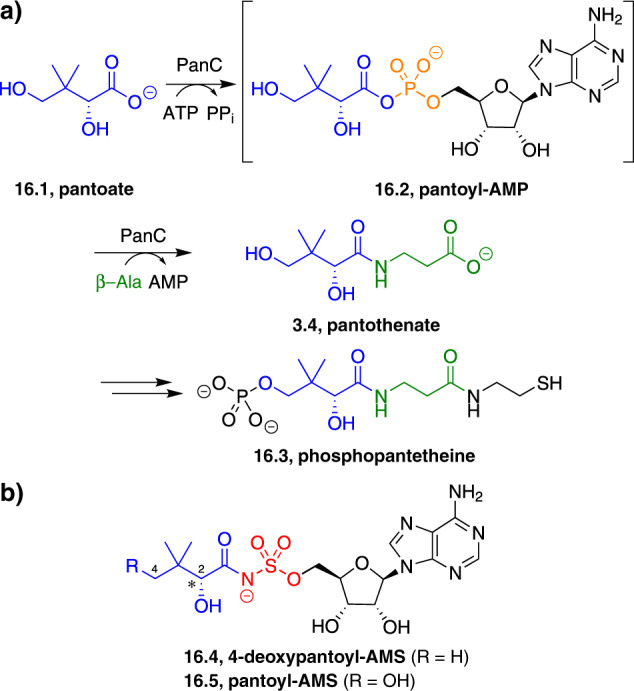
Inhibition of panthothenate synthetase (PanC). **a** PanC catalyzes adenylation of pantoate (**16.1**) to form a pantoyl-AMP intermediate (**16.2**), which reacts with a β-alanine amine nucleophile to form a pantothenate amide product (**3.4**), en route to phosphopantetheine (**16.3**). **b** A 4-deoxypantoyl-AMS inhibitor (**16.4**) mimics the pantoyl-AMP intermediate (**16.2**) but lacks the 4-hydroxy group of pantoyl-AMS (**16.5**) to avoid decomposition via lactonization of the pantoyl side chain. *Stereochemistry epimeric or as shown

Pantothenate synthetase (PanC), which shares the same fold as Class I aminoacyl-tRNA synthetases, has also been identified as a potential antibacterial target, as it is required for pantothenate biosynthesis in microorganisms, plants, and fungi, but is not found in humans [130] (Fig. 16). Pantothenate (**3.4**) is a key precursor to the phosphopantetheine moiety of coenzyme A and ACPs. The first potent, selective inhibitors of pantothenate synthetase were developed by Abell and colleagues [130, 131], using acyl-AMS analogues (e.g., (2*RS*)-**16.4**) to mimic the pantoyl-AMP reaction intermediate (**16.2**), providing sub-µM inhibition of the enzymes from *E. coli* and *Mycobacterium tuberculosis*. Subsequently, Aldrich and colleagues [132] reported a stereoselective synthesis of (2*R*)-**16.4** as well as additional analogues. Notably, all of these analogues were modified relative to the parent pantoyl-AMS inhibitor (**16.5**) to avoid lactonization of the pantoyl side chain. In the case of the 4-deoxy analogues shown, this results in loss of a putative hydrogen-bonding interaction with Gln-72, which was observed in the co-crystal structure of the *M. tuberculosis* PanC with pantoyl-AMP [48]. Moreover, none of these compounds were reported to exhibit activity in whole-cell assays.

**Fig. 17:**
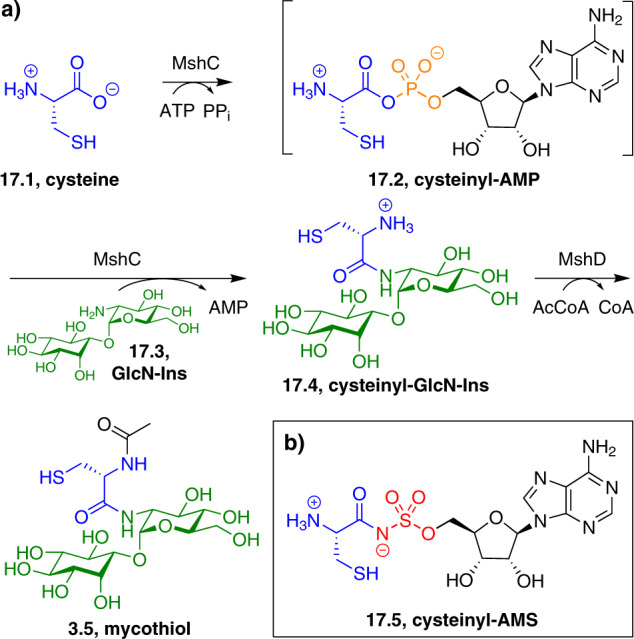
Inhibition of mycobacterial cysteine ligase (MshC). **a** MshC catalyzes adenylation of cysteine (**17.1**) to form a cysteinyl-AMP intermediate (**17.2**), which reacts with a GlcN-Ins amine nucleophile (**17.3**) to form a cysteinyl-GlcN-Ins amide product (**17.4**), en route to mycothiol (**3.5**). **b** A cysteinyl-AMS inhibitor (**17.5**) mimics the cysteinyl-AMP intermediate (**17.2**). GlcN-Ins 1-d-*myo*-inosityl-2-amido-2-deoxy-α-d-glucopyranoside

Mycobacterial cysteine ligase (MshC) is another enzyme that shares the Class I aminoacyl-tRNA synthetase fold and has been identified as a potential antibacterial target (Fig. 17) [17, 49]. MshC catalyzes an intermediate cysteine ligation step in the biosynthesis of mycothiol (**3.5**), the small-molecule thiol that is used by actinomycetes, including mycobacteria, instead of glutathione to counter oxidative stress and electrophilic toxins. Mycothiol production is thought to play a key role in mycobacterial survival and pathogenicity. Blanchard and colleagues [49] have used cysteinyl-AMS (**17.5**) to mimic the cognate cysteinyl-AMP reaction intermediate (**17.2**), enabling crystallization of MshC. Although cysteinyl-AMS would also be expected to inhibit cysteine-tRNA synthetases, which have conserved active-site residues [133], the structural information provided by this tool compound may enable development of selective MshC inhibitors in the future.

### Inhibitors of class II aminoacyl-tRNA synthetases and related enzymes

**Fig. 18:**
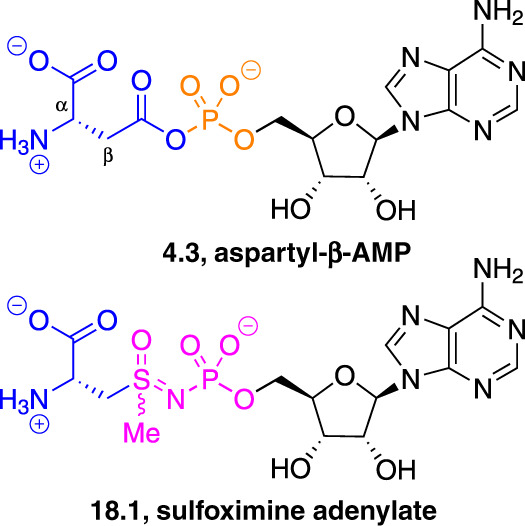
Inhibition of asparagine synthetase A. Structures of aspartyl-β-AMP reaction intermediate (**4.3**) and transition-state mimetic sulfoximine adenylate inhibitor (**18.1**)

As discussed above, class II aminoacyl-tRNA synthetases also represent potential therapeutic targets [2, 25] and aminoacyl-AMS mimics of the cognate aminoacyl-AMP reaction intermediate have been used widely in crystallographic studies [134–138]. Further, the prokaryotic enzyme AS*-*A (encoded by *asnA*) shares the same protein fold as aminoacyl-tRNA synthetases and has served as inspiration for development of inhibitors of human asparagine synthetase (ASNS), which is implicated in resistance to asparaginase therapy in acute lymphoblastic leukemia [10, 11]. Interestingly, human ASNS is structurally unrelated to prokaryotic AS-A and instead has homology to a second prokaryotic enzyme, AS-B (encoded by *asnB*), which is an N-type ATP pyrophosphatase [10, 11]. Furthermore, although AS-A directly uses ammonia for conjugation to the β-carboxylate of aspartate, AS-B and ASNS include a glutamine amidotransferase domain that hydrolyzes the side-chain amide of glutamine to generate ammonia, which is transported through a tunnel to the adenylation active site for coupling to asparate [69, 139]. Nonetheless, both mechanisms involve formation of an aspartyl-β-AMP reaction intermediate (**4.3**) (Fig. 18). Early efforts to develop ASNS inhibitors by Oda and colleagues [140] used a sulfoximine adenylate (**18.1**) as a transition-state mimic, which was a potent inhibitor of *E. coli* AS-A, and later shown to inhibit both *E. coli* AS-B and human ASNS [141, 142]. This work also led to the development of an acyl-AMS inhibitor (not shown), discussed below [143].

### Inhibitors of ANL family enzymes

#### Acyl-CoA synthetases

The first acyl-CoA synthetase inhibitors were carbonyl-reduced alkyl-AMP mimics of the cognate acyl-AMP reaction intermediate [144]. Such inhibitors have also been leveraged in structural studies of acetyl-CoA synthetase [51] as well as other acyl-CoA synthetases [145, 146]. Several bacterial acyl-CoA synthetases have been identified as promising antibacterial targets. However, as alkyl-AMP inhibitors are presumed not to be cell-penetrant, efforts to develop inhibitors of these acyl-CoA synthetases have focused primarily on the acyl-AMS design.

**Fig. 19:**
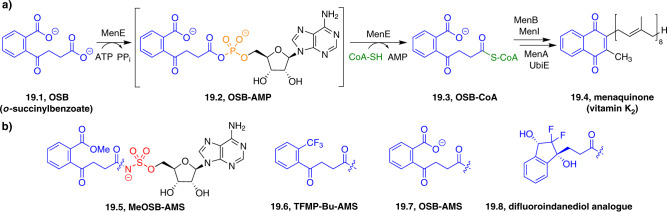
Inhibition of bacterial OSB-CoA synthetase (MenE). **a** MenE catalyzes adenylation of OSB (**19.1**) to form an OSB-AMP intermediate (**19.2**), which reacts with a CoA thiol nucleophile to form an OSB-CoA thioester product (**19.3**), en route to menaquinone (**19.4**). **b** Acyl-AMS inhibitors (19.5–19.8) mimic the OSB-AMP intermediate (**19.2**). Various modifications have been explored to replace the anionic aromatic carboxylate of OSB-AMS (**19.7**). OSB *o*-succinylbenzoate, TFMP-Bu trifluoromethylphenyl-4-oxobutyryl

The OSB-CoA (*o*-succinylbenzoate-CoA) synthetase MenE is one such target that is involved in bacterial menaquinone biosynthesis [147] (Fig. 19). Menaquinone (**19.4**; vitamin K_2_) is the sole electron transport chain quinone used in cellular respiration by Gram-positive bacteria, mycobacteria, and anaerobically growing Gram-negative bacteria. Tan, Tonge and colleagues [148] developed the first designed inhibitors of MenE, using methyl esters of OSB-AMS (**19.5**) and related sulfonyladenosines (not shown) to mimic the cognate OSB-AMP reaction intermediate (**19.2**). Contemporaneously, Mesecar and colleagues [149] reported studies of a trifluoromethyl analogue (**19.6**). In both cases, the aromatic carboxylate was masked, presumably in an effort to avoid cell penetration issues, but none of these compounds exhibited whole-cell antibacterial activity. Tan, Tonge, and colleagues [150, 151] later showed that the parent carboxylate, OSB-AMS (**19.7**), was a much more potent MenE inhibitor and exhibited modest antibacterial activity. These researchers further showed that the OSB moiety could be replaced with a difluorindanediol (**19.8**), thus removing one of the two negative charges in OSB-AMS [151, 152].

**Fig. 20:**
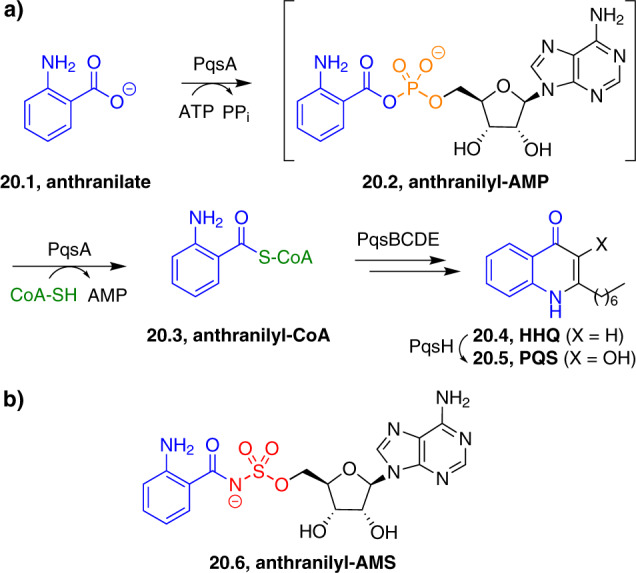
Inhibition of *P. aeruginosa* anthranilyl-CoA synthetase (PqsA). **a** PqsA catalyzes adenylation of anthranilate (**20.1**) to form an anthranilyl-AMP intermediate (**20.2**), which reacts with a CoA thiol nucleophile to form an anthranilyl-CoA thioester product (**20.3**), en route to the quinolone quorum-sensing factors HHQ (**20.4**) and PQS (**20.5**). **b** An anthranilyl-AMS inhibitor (**20.6**) mimics the anthranilyl-AMP intermediate (**20.2**). HHQ 2-heptyl-4-hydroxy-quinoline, PQS *Pseudomonas* quinolone signal = 3,4-dihydroxy-2-heptylquinoline

*Pseudomonas aeruginosa* anthranilyl-CoA synthetase (PqsA) is another ANL family member that has been identified as a potential antibacterial target [153] (Fig. 20). PqsA is involved in the biosynthesis of the quinolone quorum-sensing factors PQS (**20.5**; *Pseudomonas* quinolone signal; 3,4-dihydroxy-2-heptylquinoline) and HHQ (**20.4**; 2-heptyl-4-hydroxyquinoline), which bind the transcriptional activator MvfR (PqsR) and induce expression of a variety of virulence factors. Tan and colleagues [154] developed anthranilyl-AMS (**20.6**) and several analogues as inhibitors of PqsA that mimic the cognate anthranilyl-AMP reaction intermediate (**20.2**). However, although these compounds were potent biochemical inhibitors, they exhibited very weak activity in cell culture, which was attributed to poor cell penetration.

Plant 4-coumaroyl-CoA ligases (4CL) have also been advanced as potential targets for modulation of plant phenylpropanoid biosynthesis in biofuel applications [146, 155]. Toward this end, Watanabe and colleagues [156] recently reported evaluation of a series of 4-coumaroyl-AMS analogues (not shown) against 4CL from five plant species.

#### NRPS adenylation enzymes

**Fig. 21:**
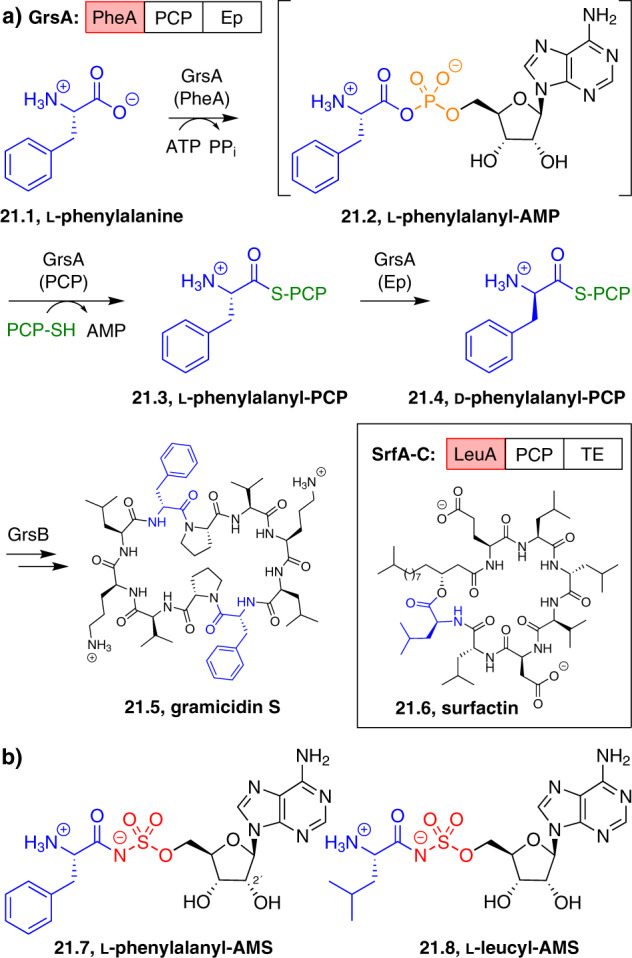
Inhibition of adenylation domains from NRPS pathways. **a** The GrsA subunit PheA domain catalyzes adenylation of l-phenylalanine (**21.1**) to form an l-phenylalanyl-AMP intermediate (**21.2**), which reacts with the GrsA PCP domain phosphopantetheine thiol nucleophile to form an acyl-PCP thioester product (**21.3**). The GrsA Ep domain then inverts the α-stereocenter of the thioester to form the d-congener (**21.4**). Peptide extension by the GrsB subunit and dimerization forms gramicidin S (**21.5**). Inset: The SrfA-C subunit LeuA domain catalyzes an analogous adenylation of l-leucine en route to surfactin (**21.6**). **b** Aminoacyl-AMS inhibitors mimic the aminoacyl-AMP intermediates of GrsA PheA (**21.7**) and SrfA-C LeuA (**21.8**), respectively. Ep epimerase, GrsA gramicidin synthetase A, LeuA leucine adenylation, PheA phenylalanine adenylation, SrfA-C surfactin synthetase C, TE thioesterase

NRPS are used in the biosynthesis of a wide range of microbial natural products, including antibiotics and virulence factors [16, 27]. NRPS adenylation domains, as well as separate adenylation enzymes, are responsible for activating amino acids and other carboxylic acids and loading them onto the NRPS assembly line via the phosphopantetheine thiol on PCP (peptidyl carrier protein or thiolation) domains (Fig. 21). By analogy to previous inhibitors of mechanistically related aminoacyl-tRNA synthetases, Marahiel and colleagues [157] first described a general approach to specific inhibition of NRPS adenylation domains using phenylalanyl-AMS (**21.7**) to target the gramicidin S synthetase A subunit (GrsA) phenylalanine adenylation domain (PheA) and leucyl-AMS (**21.8**) to target the surfactin synthetase C subunit (SrfA-C) leucine adenylation domain (LeuA). They also reported a 2′-*O*-biotinylated analogue of phenylalanyl-AMS (not shown) for affinity purification experiments. These compounds were advanced as tools to study and potentially to alter the specificity of NRPS adenylation domains. Indeed, several groups have subsequently used aminoacyl-AMS inhibitors and related affinity probes in biochemical, proteomic, structural, and reprogramming studies of NRPS adenylation domains [158–164]. In an elegant application of this inhibitor design to potential therapeutics, Marahiel and colleagues [165] have also developed d-alanyl-AMS (not shown) as an antibiotic targeting the d-alanine:d-alanyl carrier protein ligase (DltA) involved in lipoteichoic acid biosynthesis in Gram-positive bacteria.

**Fig. 22:**
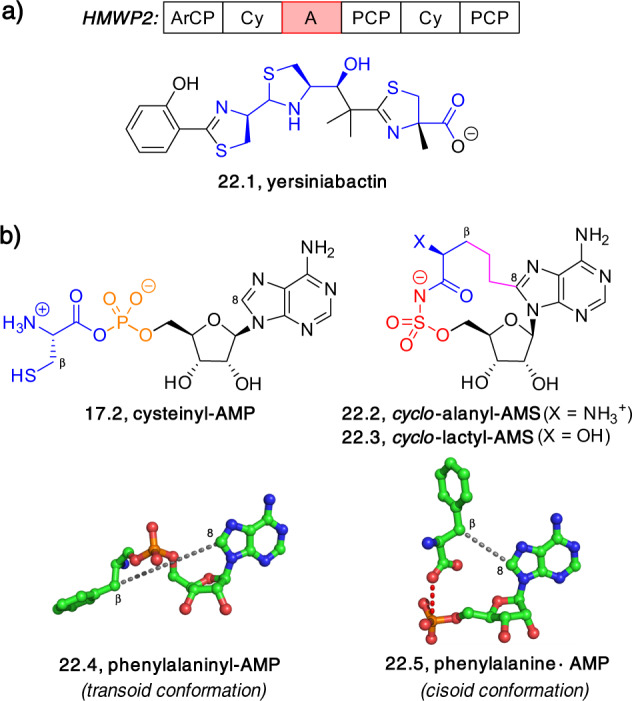
Inhibition of yersiniabactin synthethase subunit HMWP2. **a** The yersiniabactin synthethase HMWP2 subunit cysteine A domain catalyzes adenylation of l-cysteine en route to yersiniabactin (**22.1**), where it is incorporated three times (blue). **b** Both HMWP2 and cysteinyl-tRNA synthetase form the same cysteinyl-AMP reaction intermediate (17.2), but aminoacyl-tRNA synthetases bind their adenylate intermediates in extended or transoid conformations (**22.4**) (phenylalaninyl-AMP bound to phenylalanyl-tRNA synthetase, from PDB ID: 1B7Y) [218], whereas NRPS adenylation domains bind them in cisoid conformations (**22.5**) (phenylalanine and AMP bound to GrsA PheA, from PDB ID: 1AMU) [54]. The macrocyclic constraint (red) in *cyclo*-alanyl-AMS (**22.2**) enforces the cisoid conformation, resulting in selective inhibition of the HMWP2 cysteine A domain but not aminoacyl-tRNA synthetases. A *cyclo*-lactyl-AMS analogue (**22.3**) was also investigated to improve cell permeability. A adenylation domain, ArCP aroyl carrier protein, Cy condensation/cyclase, HMWP2 high molecular weight protein 2

Subsequently, Tan, Quadri and colleagues [166] developed macrocyclic acyl-AMS analogues (**22.2**) to provide specificity for NRPS amino acid adenylation domains over the corresponding aminoacyl-tRNA synthetases (Fig. 22). Recognizing that both class I and class II aminoacyl-tRNA synthetases bind their acyl-AMP reaction intermediates in relatively extended conformations (**22.4**) while ANL family enzymes bind these intermediates in “cisoid” conformations (**22.5**), they installed a linker between C8 of the adenine ring and Cβ of the amino acid to enforce the cisoid pharmacophore. These macrocycles were potent inhibitors of the cysteine adenylation domain from the *Yersinia pestis* yersiniabactin synthetase HMWP2 (high molecular weight protein 2) subunit, which is involved in the biosynthesis of the siderophore yersiniabactin (**22.1**), and did not inhibit any aminoacyl-tRNA synthetases in an in vitro translation assay. However, these zwitterionic compounds did not exhibit whole-cell activity, presumably due to poor cell penetration [167]. To address this problem, the corresponding α-hydroxyacyl macrocycles (**22.3**) and linear congeners (not shown) were also investigated, but these compounds were weaker inhibitors by two to three orders of magnitude, which was attributed to loss of a key electrostatic interaction of the α-amino group with a conserved active-site asparate residue [168].

**Fig. 23:**
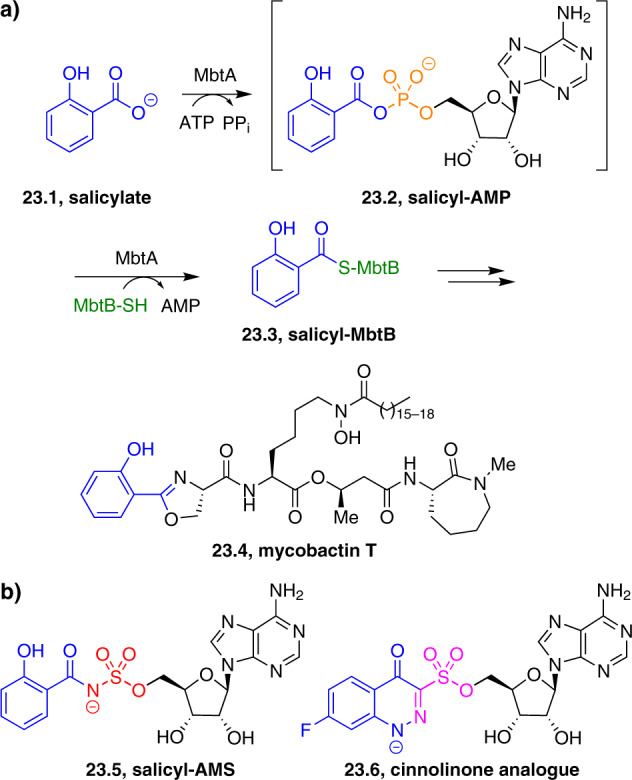
Inhibition of bacterial salicylate adenylation enzymes. **a** Salicylate adenylation enzymes, such as MbtA, catalyze adenylation of salicylate (**23.1**) to form a salicyl-AMP intermediate (**23.2**), which reacts with a phosphopantetheine thiol nucleophile on an ArCP domain, such as that in MbtB, to form a salicyl-PCP thioester product (**23.3**), en route to aryl-capped siderophores, such as *M. tuberculosis* mycobactin T (**23.4**). **b** A salicyl-AMS inhibitor (**23.5**) mimics the salicyl-AMP intermediate (**23.2**) and exhibits in vivo efficacy in a mouse model of tuberculosis. A constrained cinnolinone analogue (**23.6**) has improved pharmacological properties

Salicylate adenylation enzymes are another group of ANL family members that have attracted considerable attention as promising antibacterial targets [169] (Fig. 23). These enzymes activate the starter units in hybrid NRPS-polyketide biosynthetic pathways leading to aryl-capped siderophores. Siderophores are iron-chelating natural products that are used by pathogenic bacteria to capture iron, an essential nutrient, from the human host [28, 86, 87]. Quadri, Tan and colleagues [170] reported the first designed siderophore biosynthesis inhibitors using salicyl-AMS (**23.5**) to mimic the cognate salicyl-AMP reaction intermediate (**23.2**) of the salicylate adenylation enzymes *M. tuberculosis* MbtA, *Y. pestis* YbtE, and *P. aeruginosa* PchD, which are used in the biosynthesis of the siderophores mycobactin (**23.4**), yersiniabactin (**22.1**, Fig. 22), and pyochelin (not shown), respectively. These potent, tight-binding inhibitors also exhibited activity in cell culture as well as antibacterial efficacy in a mouse model of tuberculosis [171, 172]. However, further preclinical development was hampered by a short pharmacokinetic half-life and dose-limiting toxicity. Extensive structure–activity relationship studies by Aldrich and colleagues [7, 173–178] provided a comprehensive view of modifications in every region of the lead compound and identified analogues with improved pharmacological properties. Most recently, they have developed a series of constrained cyclic analogues in which part of the acyl sulfamate linker is replaced with a heterocycle to provide improved pharmacological properties (**23.6**) [178]. Importantly, this work established synthetic routes to diverse acyl-AMS analogues that can be applied broadly to inhibitors of other adenylate-forming enzymes. It also enabled development of a photoaffinity probe for mechanism-of-action studies of salicyl-AMS *M. tuberculosis* [179] and inspired a number of vinyl sulfonamide probes designed to trap the incoming thiol nucleophile in the second half-reaction [180–183]. Related aroyl-AMS inhibitors (not shown) have also been developed for a number of other aryl adenylation enzymes, including *Bacillus subtilis* DhbE in bacillibactin biosynthesis [184], *Bacillus anthracis* AsbC in petrobactin biosynthesis [185], mycobacterial FadD22 in phenolic glycolipid biosynthesis [186], *Acinetobacter baumannii* BasE in acinetobactin biosynthesis [187], and *E. coli* EntE in enterobactin biosynthesis [188].

**Fig. 24:**
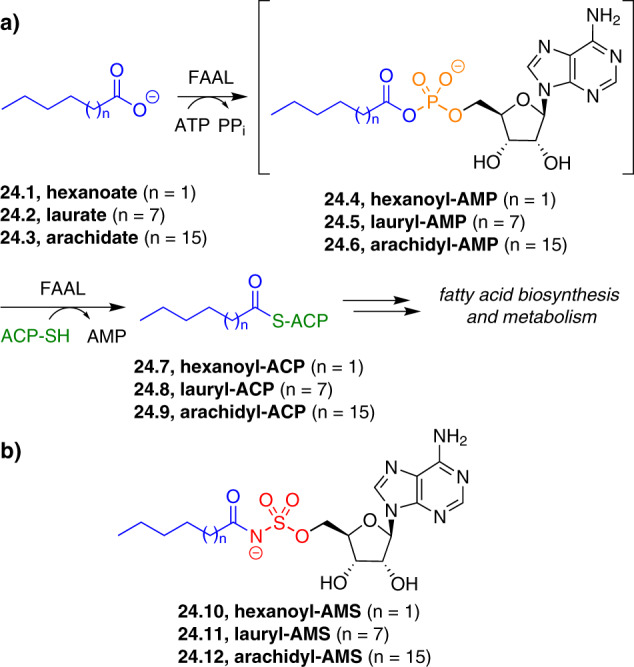
Inhibition of mycobacterial fatty acyl-AMP ligases (FAAL). **a** FAAL enzymes catalyze adenylation of fatty acids (**24.1**–**24.3**) to form fatty acyl-AMP intermediates (**24.4**–**24.6**), which react with the phosphopantetheine thiol nucleophile on an ACP domain to form fatty acyl-ACP thioester products (**24.7**–**24.9**), which are then used in fatty acid biosynthesis and metabolism. **b** Fatty acyl-AMS inhibitors (**24.10**–**24.12**) mimic the fatty acyl-AMP intermediates (**24.4**–**24.6**)

Fatty acyl-AMP ligases (FAALs) are another subset of the ANL family that have been identified as potential antibacterial targets based on their use in lipid virulence factor biosynthesis and metabolism in mycobacteria [189] (Fig. 24). These enzymes transfer the fatty acyl chain to an ACP domain of a polyketide synthetase, making them mechanistically analogous to NRPS adenylation domains [190]. A second subfamily, fatty acyl-CoA ligases (FACLs), transfer the acyl group to CoA and are analogous to acyl-CoA synthetases. Gokhale and colleagues [189] have reported a series of fatty acyl-AMS analogues (**24.10**–**24.12**) as inhibitors of both FAALs and FACLs, and these compounds exhibited modest antibacterial activity in *M. tuberculosis* and *Mycobacterium smegmatis* cell culture. Anderson and colleagues [191] have also used a related acyl-AMS inhibitor in structural studies of *M. tuberculosis* FadD32, a potential target to inhibit mycolic acid biosynthesis.

#### Firefly luciferase

**Fig. 25:**
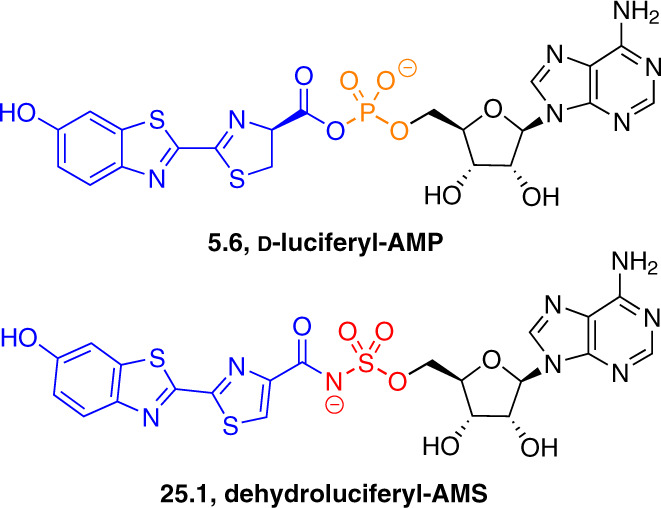
Inhibition of firefly luciferase. Structures of d-luciferyl-AMP reaction intermediate (**5.6**) and a dehydroluciferyl-AMS inhibitor (**25.1**)

Branchini and colleagues [192] developed dehydroluciferyl-AMS (**25.1**) as an oxidation-resistant analogue of the cognate luciferyl-AMP reaction intermediate (**5.6**), owing to the lack of an α-hydrogen (Fig. 25). This inhibitor was subsequently used by Kato and colleagues to study conformational changes in wild-type luciferase and a mutant known to shift luminescence from yellow–green to red [57]. This structural information was then used to design additional luciferase mutants that emitted light at different wavelengths.

### Inhibitors of ubiquitin-family E1 activating enzymes

**Fig. 26:**
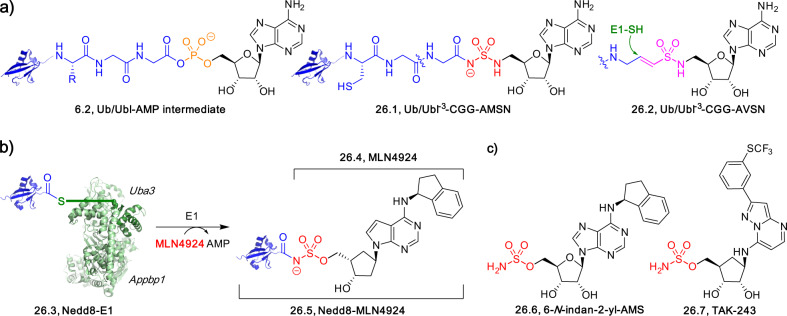
Inhibition of ubiquitin-family E1 activating enzymes. **a** Structures of Ub/Ubl-AMP reaction intermediate (**6.2**), Ub/Ubl-AMSN adenylate mimetic inhibitors (**26.1**), and Ub/Ubl-AVSN vinyl sulfonamides (**26.2**) designed to trap the E1 cysteine nucleophile in the second half-reaction. Inhibitors were synthesized by native chemical ligation, resulting in replacement of a non-conserved residue near the C terminus with cysteine. Ub/Ubl structures derived from PDB ID: 3KYC [60]. **b** Reverse reaction of the NEDD8 E1 thioester (**26.3**) with the MLN4924 prodrug (**26.4**) to form a NEDD8-MLN4924 adenylate mimetic inhibitor (**26.5**). NEDD8 and NEDD8 E1 (Uba3/Appbp1) structures derived from PDB ID: 3GZN [101]. **c** Structures of other AMS-based prodrugs (**26.6**, **26.7**). AVSN 5′-(aminodeoxy)adenosine-5′-*N*-vinyl sulfonamide, CGG Cys-Gly-Gly peptide, Ub/Ubl^–3^ = Ub/Ubl truncated by three residues at C terminus

Conjugation of Ub and other Ubl modifier proteins controls a host of cellular processes including protein homeostasis, cell cycle progression, and transcriptional regulation [5]. This complex process is catalyzed by a cascade of three enzymes, the first being an E1 activating enzyme that adenylates the C-terminal diglycine motif of the Ub/Ubl, then forms a thioester to a remote catalytic cysteine on the E1 enzyme [36] (Fig. 6). Notably, early co-crystal structures of E1s with Ubls and ATP showed the substrates bound in the adenylation active site [193, 194], in contrast to other adenylate-forming enzymes where the acyl-AMP reaction intermediate is typically observed. Moreover, the catalytic cysteine residue was over 30 Å away from the adenylation active site [193–195]. To probe the molecular mechanism of E1 catalysis, Tan, Lima and colleagues [60, 196] used native chemical ligation of truncated Ubls with synthetic peptides to generate Ubl-AMSN sulfamide mimics (**25.1**) of the Ubl-AMP reaction intermediates (**6.2**) and Ubl-AVSN vinyl sulfonamide probes (**25.2**) designed to trap the catalytic cysteine nucleophile covalently (Fig. 26a). Biochemical studies showed that the SUMO (small ubiquitin-like modifier) and Ub probes were specific for their respective E1s. Structural studies then revealed that nucleophilic addition in the second half-reaction was accompanied by a 130° rotation of the cysteine-containing domain as well as remodeling of nearly half of the residues in the active site. This domain alternation is reminiscent of conformational changes in the ANL family [35]. Importantly, this work extended the applications of the acyl-AMS inhibitor design platform beyond small-molecule substrates to proteins and also highlighted the power of these inhibitors to elucidate mechanistic insights that had resisted classical biochemical efforts for over 30 years.

Notably, Soucy and colleagues [197] at Millenium Pharmaceuticals discovered a potent small-molecule inhibitor of the NEDD8 E1 activating enzyme, designated MLN4924 (**26.4**; pevonedistat), derived from a high-throughput screening and medicinal chemistry campaign (Fig. 26b). This compound has advanced to Phase I and II clinical trials for a variety of cancers. Contemporaneously with the mechanistic studies of Tan, Lima and colleagues [196] above, Brownell and colleagues [101] at Millenium discovered that MLN4924 actually acted as a prodrug that reacted with the NEDD8 E1 thioester (**26.3**) to form a NEDD8-MLN4924 inhibitor (**26.5**) via a reverse reaction. This inhibitor is analogous to Ubl-AMSN, albeit with several modifications that confer specificity for formation by the NEDD8 E1. They further showed that an AMS analogue having a indane at the adenine-6-*N*-position (**26.6**) formed analogous adducts with NEDD8, SUMO, and Ub catalyzed by the corresponding E1 enzymes (Fig. 26c). This suggested that this AMS-based prodrug strategy might be applicable to other E1 enzymes and, recently, a Ub E1-selective inhibitor, TAK-243 (**26.7**; MLN7243) was developed and has advanced to Phase I clinical trials [198]. Building upon this work, Statsyuk and colleagues [199, 200] have used this strategy to develop activity-based AMS-alkyne probes (not shown) of Ub/Ubl signaling pathways.

**Fig. 27:**
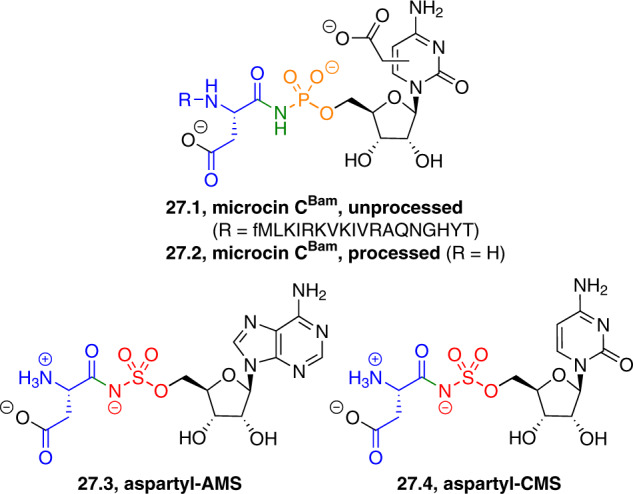
Microcin-like Trojan horse antibiotics from *B. amyloliquefaciens*. **a** A microcin C-like antibiotic from *B. amyloliquefaciens* (microcin C^Bam^) has a C-terminal cytidyl-phosphoramidate (**27.1**) and is presumed to be imported into target cells by peptide transporters, then proteolyzed by intracellular peptidases to afford the processed cytidyl-phosphoramidate inhibitor (**27.2**). **b** Aspartyl-AMS (**27.3**) and aspartyl-CMS (**27.4**) were designed to mimic processed *E. coli* microcin C7 and *B. amyloliquefaciens* microcin C, respectively, and both were shown to inhibit aspartyl-tRNA synthetase. fMLKIRKVKIVRAQNGHYT N-formyl peptide

Bacterial MccB enzymes are structurally related to eukaryotic E1 enzymes and catalyze adenylation reactions at the C terminus of peptide substrates during the biosynthesis of microcin Trojan horse antibiotics [64]. Recently, Severinov and colleagues [65] discovered a novel microcin-like biosynthetic pathway in *B. amyloliquefaciens* in which the peptide substrate is cytidylated rather than adenylated (**27.1**) (Fig. 27). They used aspartyl-AMS (**27.3**) and the corresponding cytidylate analogue (**27.4**) as analogues of processed microcins and demonstrated that both inhibited aspartyl-tRNA synthetase.

### Inhibitors of biotin protein ligases

**Fig. 28:**
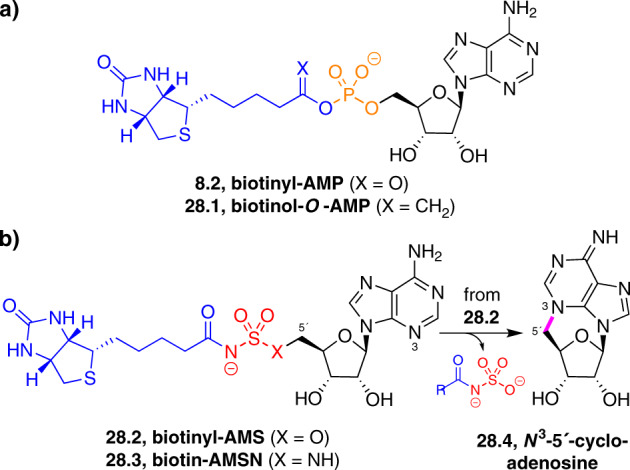
Inhibition of biotin protein ligases. **a** Structures of biotinyl-AMP reaction intermediate (**8.2**) and carbonyl-reduced intermediate-mimetic biotinol-*O*-AMP inhibitor (**28.1**). **b** Structures of adenylate-mimetic inhibitors biotinyl-AMS (**28.2**) and biotinyl-AMSN (**28.3**), and decomposition of **28.2** via formation of *N*^3^–5′-cycloadenosine (**28.4**)

Inhibitors of the *E. coli* biotin repressor BirA were first developed by Brown and Beckett [201] as tool compounds to study enzyme structure and function (Fig. 28). They demonstrated that both biotinyl-AMS (**28.2**) and the carbonyl-reduced intermediate analogue biotinol-*O*-AMP (**28.1**) were tight-binding inhibitors that could be used to activate BirA dimerization and binding to the *bioO* operon [201, 202].

Subsequently, Aldrich and colleagues [203–206] pursued a homologous *M. tuberculosis* biotin protein ligase as a potential antibacterial target, based on its role in regulating fatty acid biosynthesis and metabolism via biotinylation of acyl-CoA carboxylases and pyruvate carboxylase, respectively. Noting spontaneous decomposition of biotinyl-AMS (**28.2**) via cyclization to *N*^3^-5′-cycloadenosine (**28.4**) [104], they investigated the corresponding sulfamide, biotinyl-AMSN (**28.3**), which does not undergo this decomposition pathway and showed that it is a potent biochemical inhibitor and exhibits promising in vitro antibacterial activity against *M. tuberculosis*, including multidrug-resistant strains [203].

With Schnappinger and colleagues [207], they further showed that biotinyl-AMSN (**28.3**) had synergistic activity with two first-line tuberculosis drugs in a mouse model of *M. tuberculosis* infection. However, the compound was reported to undergo cleavage of the acyl sulfamide in vivo. Furthermore, they discovered resistant mutants in in vitro studies, which overexpressed a dioxygenase Rv3406. Drawing upon their extensive experience with salicyl-AMS analogues discussed above, Aldrich and colleagues [204, 206] carried out analogous structure–activity relationship studies of biotinyl-AMSN to improve its pharmacological properties. Notably, they installed a 5′-methyl group on the ribose fragment to avoid oxidation by Rv3406 [205]. This sets the stage for further preclinical evaluation of *M. tuberculosis* biotin protein ligase as a new antitubercular target.

### Inhibitors of *N*-type ATP Pyrophosphatases

**Fig. 29:**
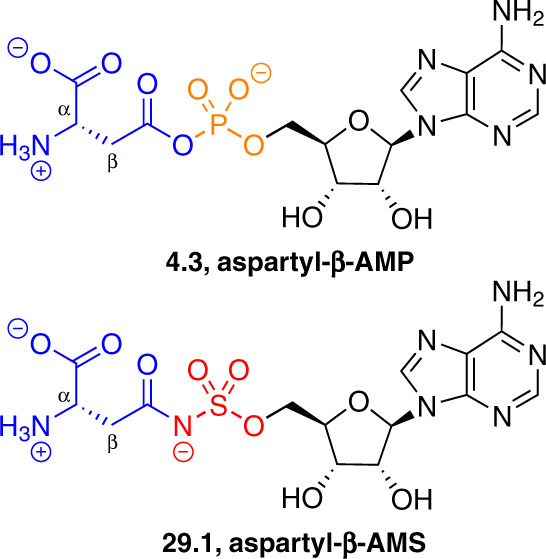
Inhibition of human asparagine synthetase (ASNS). Structures of aspartyl-β-AMP reaction intermediate (**4.3**) and adenylate-mimetic inhibitor aspartyl-β-AMS (**29.1**)

Human asparagine synthetase (ASNS) is upregulated in asparaginase-resistant forms of acute lymphoblastic leukemia and has been advanced as a potential therapeutic target [10, 11]. Building upon the earlier work of Oda and colleagues [140] on sulfoximine-based inhibitors of bacterial AS-A (Fig. 18), Richards and colleagues [143] explored aspartyl-β-AMS (**29.1**) as a more synthetically tractable scaffold to facilitate anticipated medicinal chemistry efforts (Fig. 29). Although subsequent efforts turned back to the sulfoximine adenylate scaffold [142], this work demonstrated the effectiveness of the acyl-AMS inhibitor platform for targeting an *N*-type ATP pyrophosphatase and opens the door to applications of this approach to other enzymes in this family.

## Conclusions

The adenylate-forming enzyme superfamily includes numerous attractive targets that are of both fundamental and therapeutic interest. Because of the mechanistic conservation across the superfamily, many of these targets have been inhibited effectively using acyl-AMS mimics of the cognate acyl-AMP reaction intermediate, and certainly one anticipates that other targets should be amenable to this same approach, independent of protein fold (e.g., carbamoyltransferases, NRPS-independent siderophore synthetases, BioW acyl-CoA synthetases).

However, although the acyl-AMS platform typically provides potent inhibitors, these compounds have several limitations that are particularly associated with the acyl sulfamate moiety, including potential hydrolysis, decomposition by *N*^3^-5′-cyclonucleoside formation, and a negatively charged nitrogen that may limit cell penetration and other pharmacological properties. Thus, although several of these compounds have advanced to in vivo proof-of-concept studies in mouse models, medicinal chemistry efforts are typically required to identify optimized lead compounds suitable for preclinical development. Fortunately, extensive structure–activity relationship studies have already been carried out for several targets. As a result, synthetic routes are now well-established to modify any region of the acyl-AMS scaffold. In conjunction with the availability of co-crystal structures for many of these enzymes with acyl-AMP intermediates or acyl-AMS inhibitors, this should facilitate development of analogues with improved or retained biochemical potency and improved pharmacological properties. In a related vein, it will be interesting to see whether the AMS-based prodrug strategy discovered in the context of the NEDD8 E1 inhibitor MLN4924 [101, 197] may be applicable to other classes of adenylate-forming enzymes, as these sulfonyladenosines lack the problematic acyl sulfamate moiety, which is only formed in situ by the target enzyme itself.

Notably, the highly variable cell penetration observed for various acyl-AMS inhibitors has also inspired prospective analysis of the relationships between chemical structure and bacterial penetration in an activity-independent manner [167]. This is a poorly understood area that represents a major current obstacle in antibacterial drug discovery [208–211]. Initial work by Tan and colleagues [167] using a panel of acyl-AMS compounds established the feasibility of this approach in developing predictive models for compound accumulation in a variety of bacteria and this approach was recently extended by Hergenrother and colleagues [212] to a larger collection of diverse structures. These efforts should facilitate development of acyl-AMS analogues with improved bacterial penetration, in addition to the biochemical potency and pharmacological properties mentioned above.

In summary, natural product-inspired acyl-AMS have provided a powerful entry into inhibition of adenylate-forming enzymes. This straightforward inhibitor design platform has proven effective for a wide range of biologically and structurally diverse targets. This approach promises to continue providing tool compounds for fundamental biochemical and cellular studies, as well as therapeutic lead compounds to address a variety of human diseases.
